# Supplementary material for: On the stability of canonical correlation analysis and partial least squares with application to brain-behavior associations
Source: Commun Biol. 2024 Feb 21;7:217. doi: 10.1038/s42003-024-05869-4 (PMC11245620; doi:10.1038/s42003-024-05869-4)
Supplement: Supplementary file 2 — Supplementary Information [file 42003_2024_5869_MOESM2_ESM.pdf]

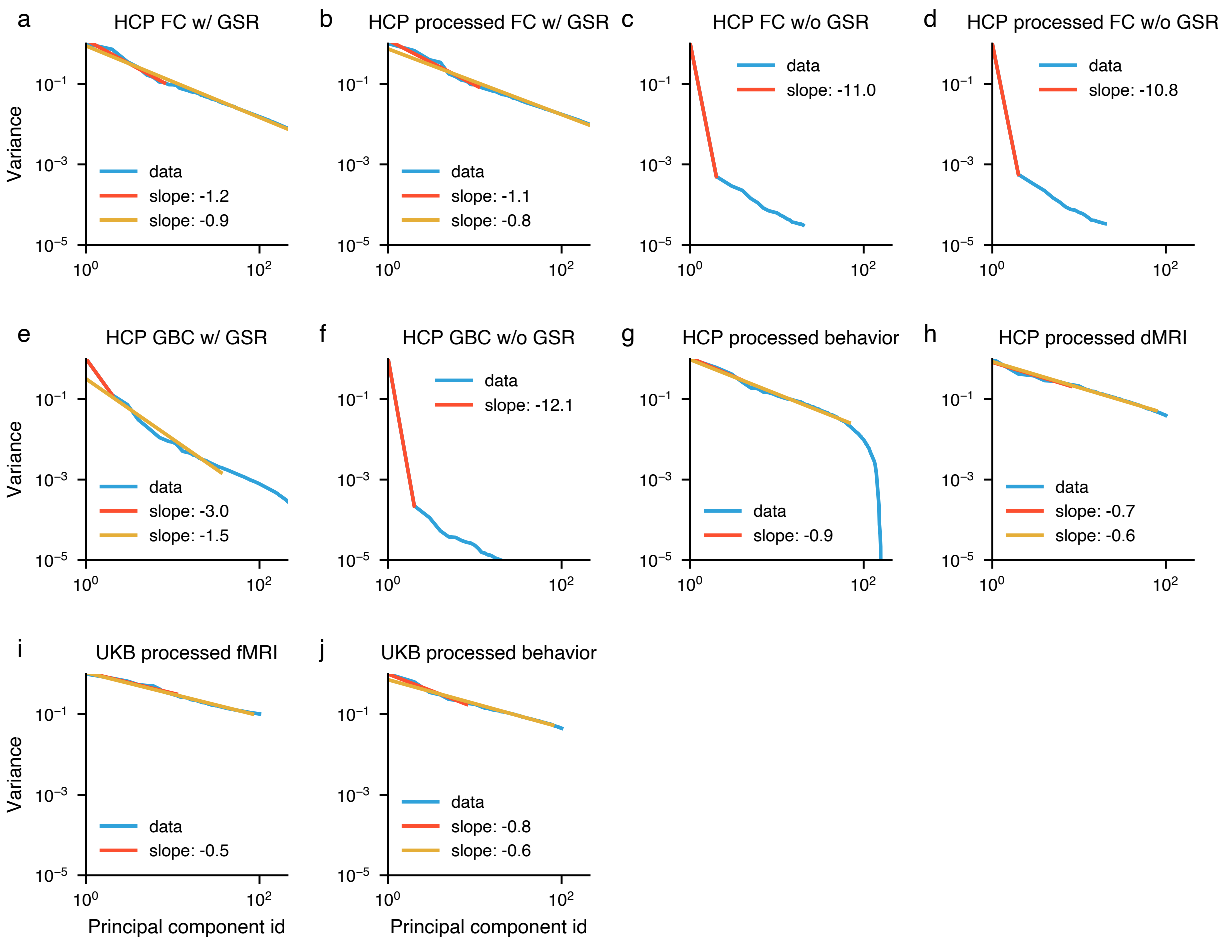

Supplementary Figure 1. Supplementary analyses of empirical data. Decay constants of principal component spectra in empirical data. Decay constants are estimated as the slope in a linear regression for the logarithm of the explained variance on the logarithm of the associated principal component number. We include enough components into the linear regression as necessary to explain either 30 % (red) or 90 % (yellow) of the variance. Where the two resulting slopes coincide only one is shown. Shown are decay constants for the following data matrices: **a)** HCP functional connectivity and **b)** HCP functional connectivity after preprocessing for CCA / PLS, both based on 951 subjects. **c)** HCP functional connectivity for 877 subjects where global signal was not regressed out, and **d)** HCP functional connectivity of 877 subjects where global signal was not regressed out after preprocessing for CCA / PLS. **e)** HCP global brain connectivity (GBC), i. e. the sum across rows of the parcel $\times$ parcel functional connectivity matrix (951 subjects) and **f)** HCP GBC where global signal was not regressed out (877 subjects). **g)** HCP behavioral data of 951 subjects after preprocessing for CCA / PLS **h)** HCP diffusion MRI structural connectivity blueprints of 1020 subjects after preprocessing for CCA / PLS. **i)** UK Biobank fMRI of 20000 subjects after preprocessing for CCA / PLS, **j)** UK Biobank behavioral measures of 20000 subjects after preprocessing for CCA / PLS.

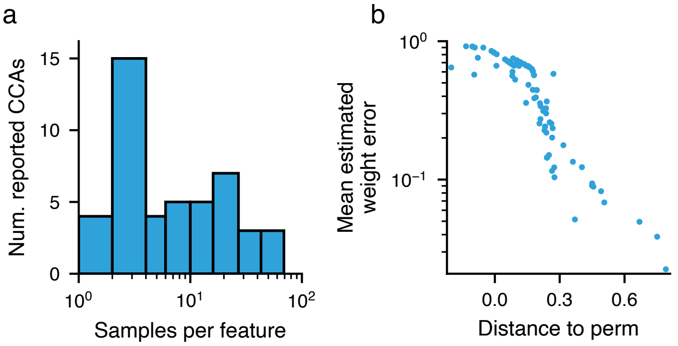

Supplementary Figure 2. Supplementary results related to analysis of prior literature. a) Typical number of samples per feature in brain-behavior CCAs. Studies using CCA to analyze brain-behavior relationships often used less than 5 samples per feature. Note that we here considered the number of features that entered into the CCA analysis, which, after preprocessing, can be considerably less than the "raw" number of features. **b)** Distance from null in *samples-per-feature vs observed correlation* plot predicts weight error. A linear model was fit to the simulated, permuted data shown in Fig. 6a and for each reported CCA the orthogonal distance to the fit-line was measured and is shown here on the x-axis, with positive values indicating deviations towards the top-right corner of Fig. 6a. The mean estimated weight error for the reported CCAs is smaller the farther away from the permuted data the CCA lies in the top-right part of the plot.

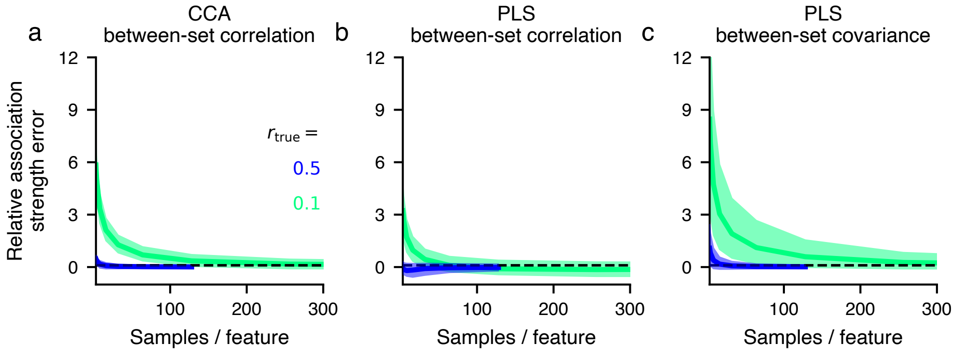

Supplementary Figure 3. Association strength error converges to 0. In-sample estimates of the relative association strength error converge to 0, for both CCA (**a**) and PLS regardless of if the association strength a is measured as between-set correlation (**b**) of between-set covariance (**c**). Relative association strength error is computed as $(\hat{a} - a_{\text{true}})/a_{\text{true}}$ where $\hat{a}$ is an estimate from a given collection of observations, and a_{true} referes to the true value. Curves show mean and 95% confidence intervals of CCA/PLS estimates across 100 draws of collections of observiations with a given sample size from 25 different generative models with the indicated r_{true} but varying true (population) weight vectors (see Methods). X and Y feature space dimensionality was 8. The simulations used for this figure are the same as those used for Fig. 2.

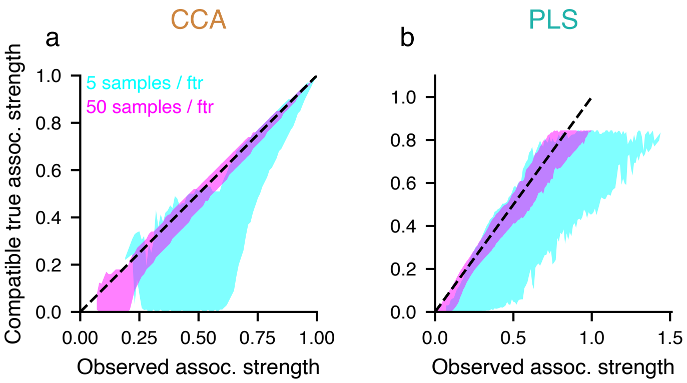

Supplementary Figure 4. A wide range of true association strengths is compatible with a given observed association strength. Synthetic datasets were generated where the true between-set correlation was varied from 0 to 0.99 in steps of 0.01 and analyzed with **a)** CCA, **b)** PLS. We investigated 4, 8, 16, 32, 64 and 128 features per set, set up 10 different covariance matrices with differing true weight vectors for each number of features and true between-set correlation, and drew 100 repeated datasets from each corresponding normal distribution. For every CCA and PLS we recorded the observed association and grouped them in bins of width 0.01. The plots show 95 % confidence intervals of the true association strength that were associated with a given observed association strength. Notably, apart from the very strongest observed association strengths which indicate an almost equally strong true between-set correlation, compatible true association strengths can be markedly lower, down to essentially 0, when the number of used samples per feature is low.

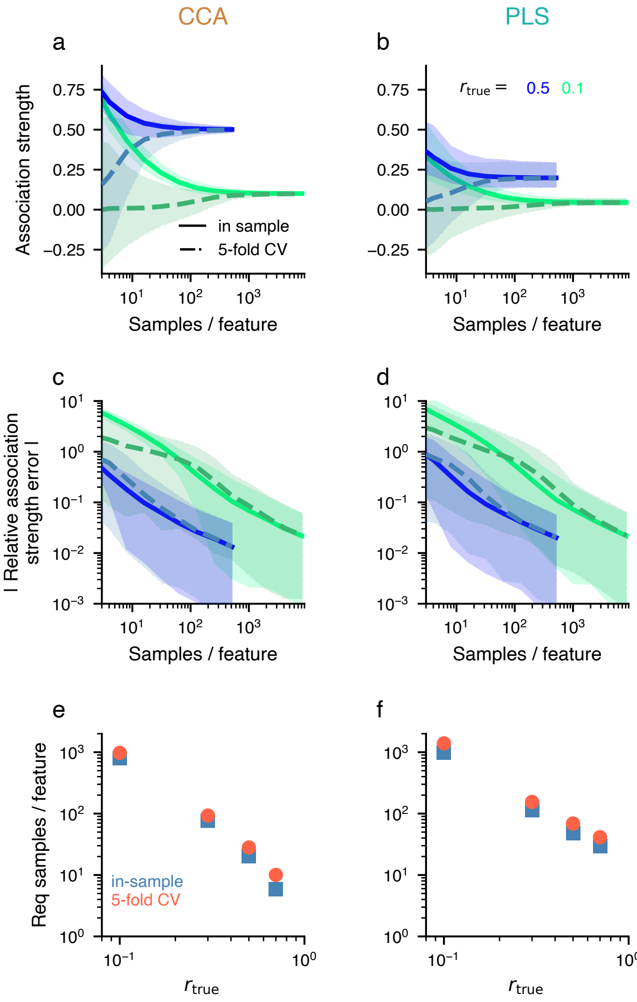

Supplementary Figure 5. Cross-validated estimation of association strength. a-b) In contrast to in-sample estimates, cross-validated estimates of between-set association strengths (i. e. between-set correlations) underestimate the true value of the between-set correlation r_{true}. We here used 5-fold cross-validation. **c-d)** The absolute value of the relative estimation error is similar for in-sample and cross-validated estimates. Shaded areas in a-d) show 95% confidence intervals across 10 covariance matrices encoding CCA/PLS solutions with the indicated r_{true} but different weight vectors, and 100 draws of collections of observations with the indicated sample sizes (x-axis) from the multivariate normal distributions associated with these covariance matrices. **e-f)** Required sample size to achieve a target error level (here: 10 %) are similar when calculated from in-sample and out-of-sample estimates. We used an X and Y feature space dimensionality of 8 in this figure.

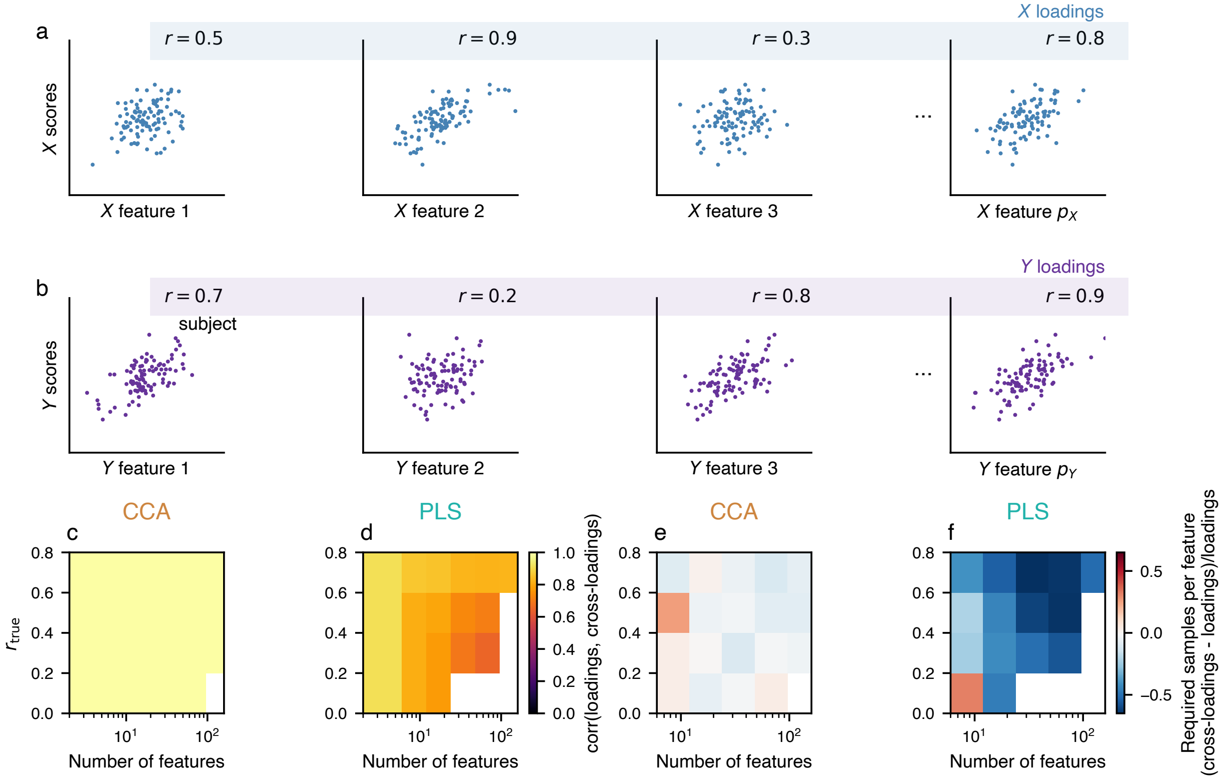

Supplementary Figure 6. Loadings and cross-loadings. a-b) Loadings are defined as Pearson correlations, r, (across observations) of a feature with the CCA/PLS scores. The loadings vector contains these correlations for all variables. Apart from the illustrated loadings, *cross-loadings* in which scores of one set are correlated with the original features of the other set can also be computed. **c-d)** Color values represent Pearson correlation between true loadings and true cross-loadings. The shown correlations were averaged across generative models different assumed weight vectors. **c)** In CCA, true loadings and true cross-loadings were collinear. **d)** For PLS, they were strongly correlated. **e-f)** For PLS cross-loadings provide more stable estimates of feature profiles than loadings. For both loadings and cross-loadings we calculated the required number of samples per feature to obtain less than 10% error. Shown here color-coded is their the difference, i. e. the required sample-per-features for cross-loadings minus for loadings, divided by the required samples-per-feature for loadings. **e)** Relative differences were small for CCA. **f)** However, for PLS fewer observations were required with cross-loadings than with loadings to obtain the same error level. r_{true} indicates the true between-set correlations used in each respective simulation. 25 covariance matrices with different true weight vectors (10, due to computational expense, in the case of PLS with r_{true}=0.7 and number of features=128) were used to instantiate generative models.

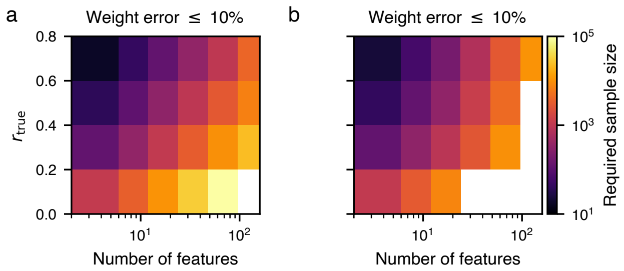

Supplementary Figure 7. Sample sizes required to obtain less than 10% weight error are shown for CCA (**a**) and PLS (**b**), depending on the assumed true correlation r_{true} and the total number of features in the data. Unless r_{true} is high, 100s to 1000s of samples are required. r_{true} denotes the assumed true between-set correlation. Required sample sizes were averaged across 25 generative models with different assumed weight vectors (10, due to computational expense, in the case of PLS with r_{true}=0.7 and number of features=128).

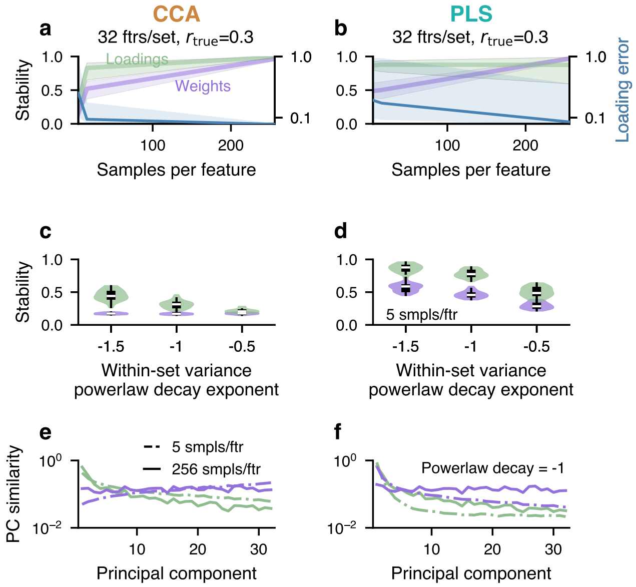

Supplementary Figure 8. Stability and PC similarity of weights and loadings. Stability of loadings was quantified with pairwise Pearson correlation of loadings from independent collection of samples, averaged over 25 generative models with different assumed weight vectors. Loading error was averaged across 100 draws of collections of observations from each of these 25 generative models. **a-b)** PLS loadings exhibit very high stability despite large loading error at lower sample sizes, in contrast to CCA. Shaded areas show 95% confidence intervals. **c-d)** The steeper the within-set variance spectrum the more stable were loadings for both CCA and PLS, and weights for PLS. Thin black bars indicate 95% confidence intervals, thick black bars the interquartile range and the white bar represents the median. **e-f)** CCA loadings resembled dominant principal component (PC) axes, while CCA weights for small sample sizes resembled more low-variance PC-axes. In contrast, PLS weights and loadings both exhibited strong bias toward dominant PC axes. r_{true} is the true between-set correlation assumed in the generative model.

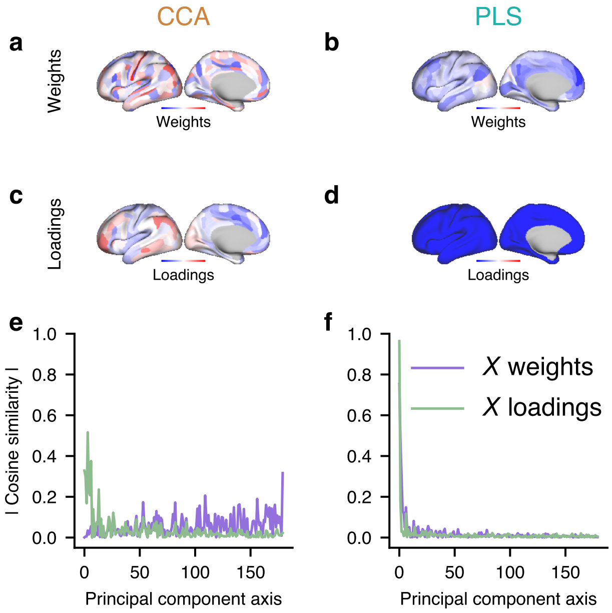

Supplementary Figure 9. Weights vs loadings in real data. Using 180 fMRI-GBC features and the 5 dominant behavioral principal components as input to CCA / PLS we here illustrate GBC weights and loadings. **a** CCA weights, **b** PLS weights, **c** CCA loadings, and **d** PLS loadings. Note the relative noisiness of CCA weights. **e-f** shows a decomposition of weights and loadings into principal components, illustrating that CCA weights overlap more with low-variance PC-axes, while CCA loadings, as well as PLS weights and loadings overlap more with dominant PC-axes.

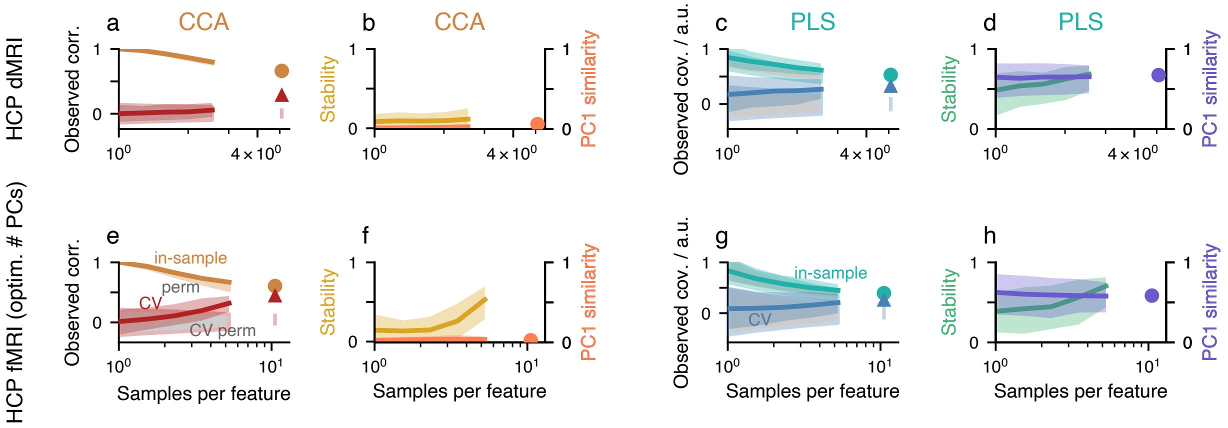

Supplementary Figure 10. Additional CCA and PLS analyses of HCP data. Layout is similar to first row in Fig. 5. **a-d**) HCP dMRI data was related to behavioral and demographic data. Overall, CCA and PLS behave similarly using dMRI compared to fMRI data (Fig. 5a-d). p-values in **a** and **c** were 0.001 and 0.001, respectively. **e-h)** Re-analysis of HCP fMRI vs behavior data with optimized number of principal components. Format is identical to Fig. 5. The only difference is the number of principal components retained for analysis: whereas in Fig. 5 100 principal components were used for both datasets, in agreement with previous studies of HCP data, here we chose the number of principal component with a "max-min detector". As the algorithm provided multiple values for the optimal number of components p_X (neuroimaging data) and p_Y (behavioral and demographic data), we selected here the pair that minimized $p_X + p_Y$. The optimized values were $p_X = 59$ and $p_Y = 31$, along with 13 between-set modes (we only consider the first one here). p-values for CCA and PLS were, respectively, 0.001 and 0.004. While the results are very similar to Fig. 5, (i) the observed between-set correlations in **e)** appear to have stabilized more and are lower than in Fig. 5a, (ii) in-sample and cross-validated association strengths are more similar here in panels **a)** and **c)** than in Fig. 5, and (iii) weight similarities in **b)** and **d)** are higher than in Fig. 5. Altogether results seem to have converged more with the same sample size. This demonstrates the potential benefit of dimensionality reduction for CCA and PLS. Shaded areas indicate 95% confidence intervals across subsample pairs and (where applicable) 1000 permutations. All analyses were performed with 100 randomly drawn subsamples of varying sizes (x-axis). For each subsample size and repetition, we created two non-overlapping sets of subjects and calculated weight stability using these non-overlapping pairs.

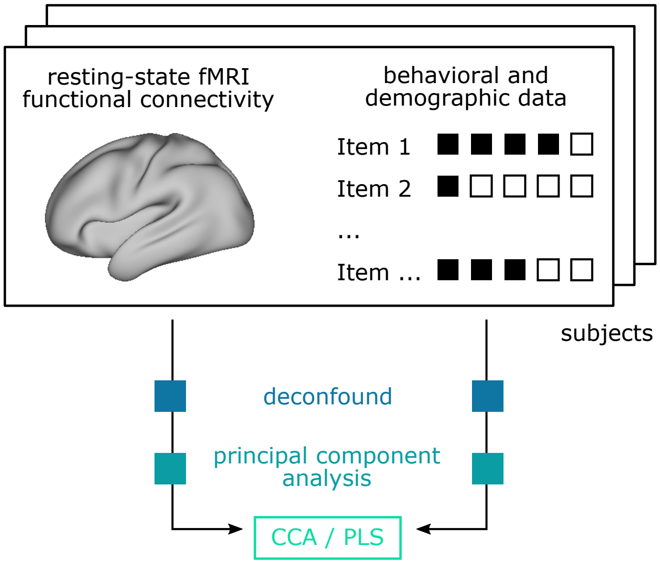

Supplementary Figure 11. HCP data analysis workflow. Resting-state functional connectivity data and behavioral and demographic data from corresponding subjects were separately deconfounded, reduced to 100 principal components and then analyzed with CCA and PLS.

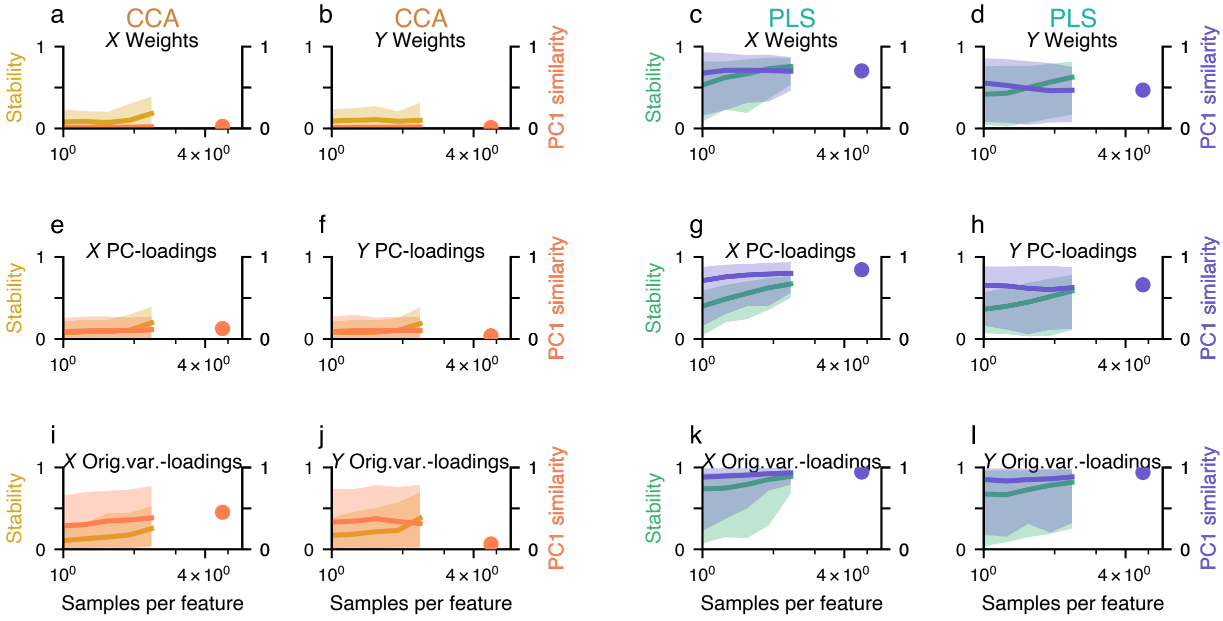

Supplementary Figure 12. HCP fMRI CCA/PLS results for X and Y separately. For conciseness, sample size dependences of weights, PC loadings and original-variable loadings were collapsed into one quantity in Fig. 5 and Supplementary Fig. 14. Here, X and Y weights and loadings are shown separately. Shaded areas indicate 95% confidence intervals across subsample pairs and (where applicable) 1000 permutations. All analyses were performed with 100 randomly drawn subsamples of varying sizes (x-axis). For each subsample size and repetition, we created two non-overlapping sets of subjects and calculated weight stability using these non-overlapping pairs.

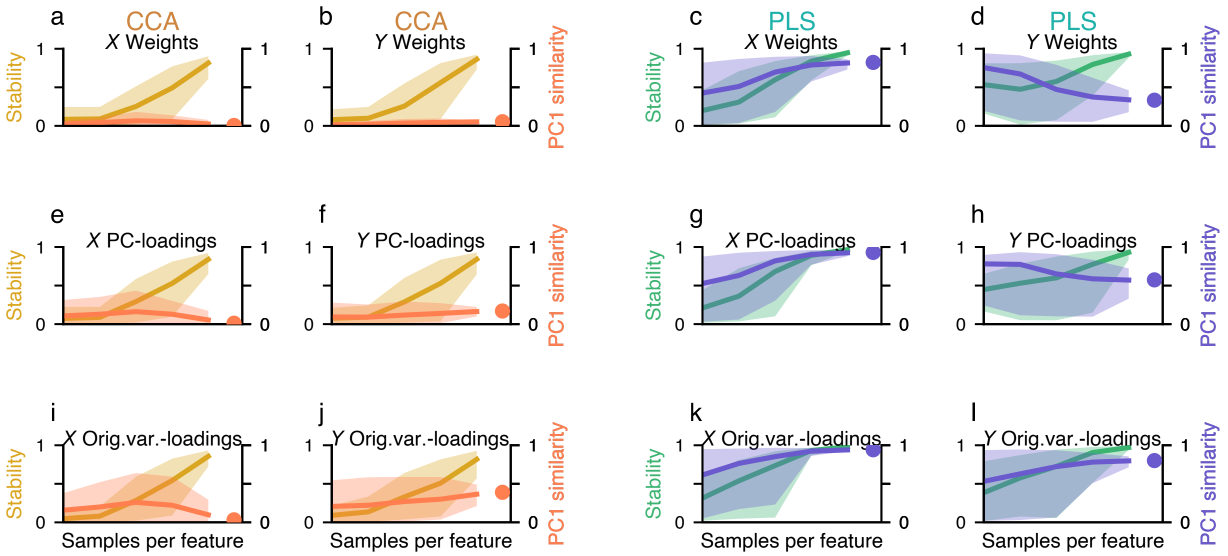

Supplementary Figure 13. UKB fMRI CCA/PLS results for X and Y separately. For conciseness, sample size dependences of weights, PC loadings and original-variable loadings were collapsed into one quantity in Fig. 5 and Supplementary Fig. 14. Here, X and Y weights and loadings are shown separately. Shaded areas indicate 95% confidence intervals across subsample pairs and (where applicable) 1000 permutations. All analyses were performed with 100 randomly drawn subsamples of varying sizes (x-axis). For each subsample size and repetition, we created two non-overlapping sets of subjects and calculated weight stability using these non-overlapping pairs.

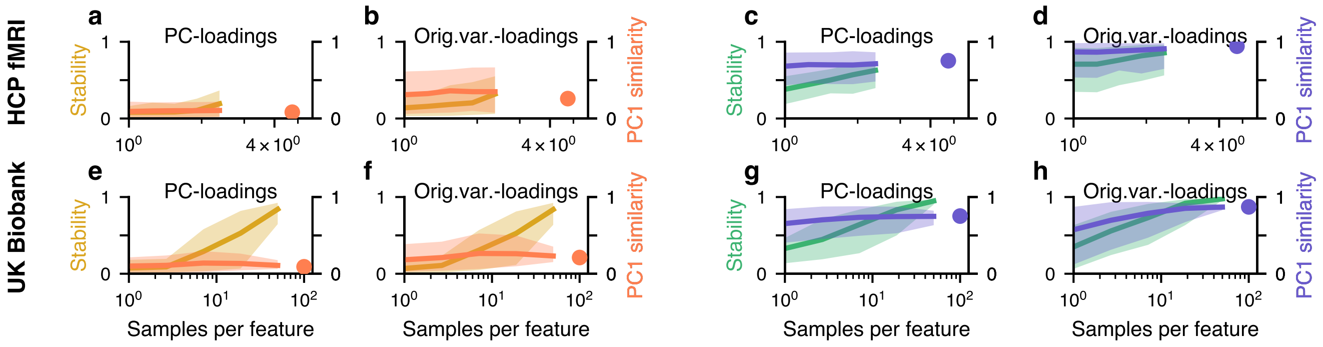

Supplementary Figure 14. Loadings in HCP fMRI and UKB. Continuation of Fig. 5. Both PC-loadings (calculated as the correlation across observations between CCA/PLS scores and PCs of the data matrices) and original-variable-loadings (calculated as the correlation across observations between CCA/PLS scores and the columns of the data matrices) show a similar pattern as weights (compare Fig. 5), with loadings being slightly more similar to PC1 than weights. Stability and PC1 similarity were calculated according to Eqs. 17 and 18, respectively. Shaded areas indicate 95% confidence intervals across subsample pairs and (where applicable) 1000 permutations. All analyses were performed with 100 randomly drawn subsamples of varying sizes (x-axis). For each subsample size and repetition, we created two non-overlapping sets of subjects and calculated weight stability using these non-overlapping pairs.

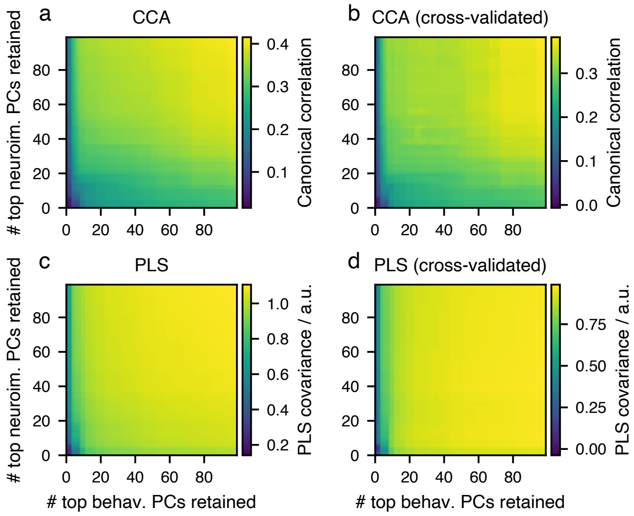

Supplementary Figure 15. CCA and PLS association strength in UKB depending on retained number of principal components. a) In-sample and **b)** cross-validated association strength for CCA, measured as between-set correlation. **c)** In-sample and **d)** cross-validated association strength for PLS, measured as between-set covariance.

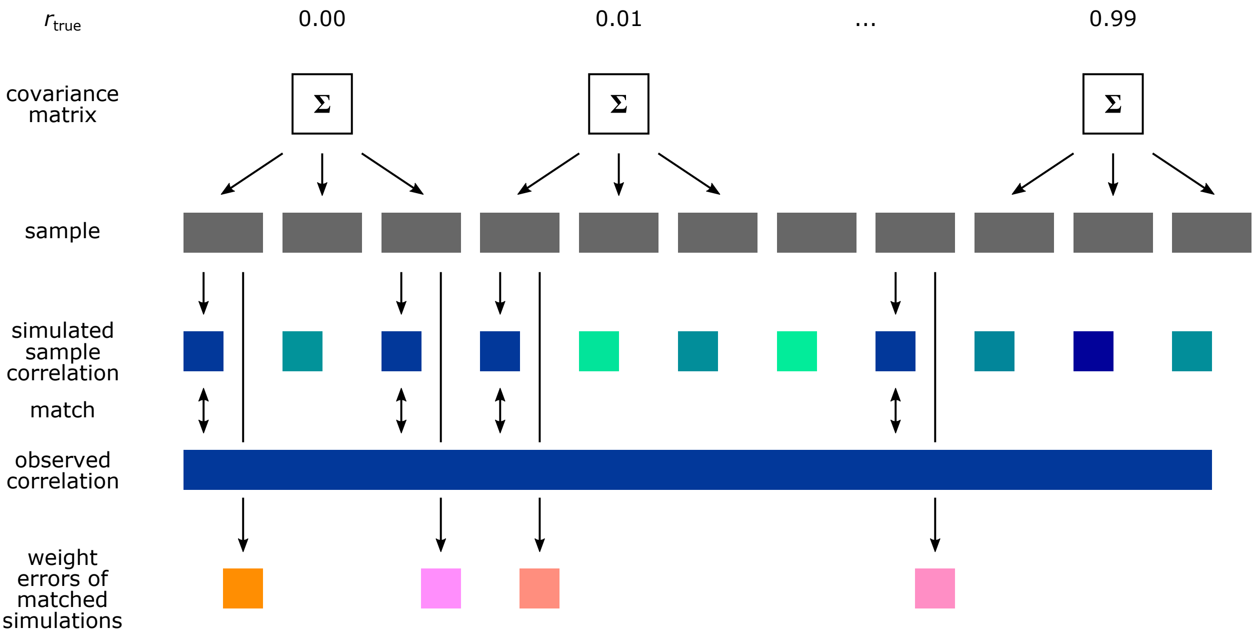

Supplementary Figure 16. Schematic for estimating weight errors for published CCA results. For each CCA from the literature in our database, synthetic data for CCA is generated with matching number of samples and features. 100 collections of observations are drawn for each assumed ground-truth between-set correlation r_{true} of 0.00, 0.01 ... 0.99. In each generated dataset the between-set correlation is estimated and if it is close to the value in the reported CCA, the weight error for the synthetic dataset is recorded. The distribution of recorded weight errors across assumed ground-truth between-set correlations and repetitions of the whole process is shown in Fig. 6b and its mean in Fig. 6a.

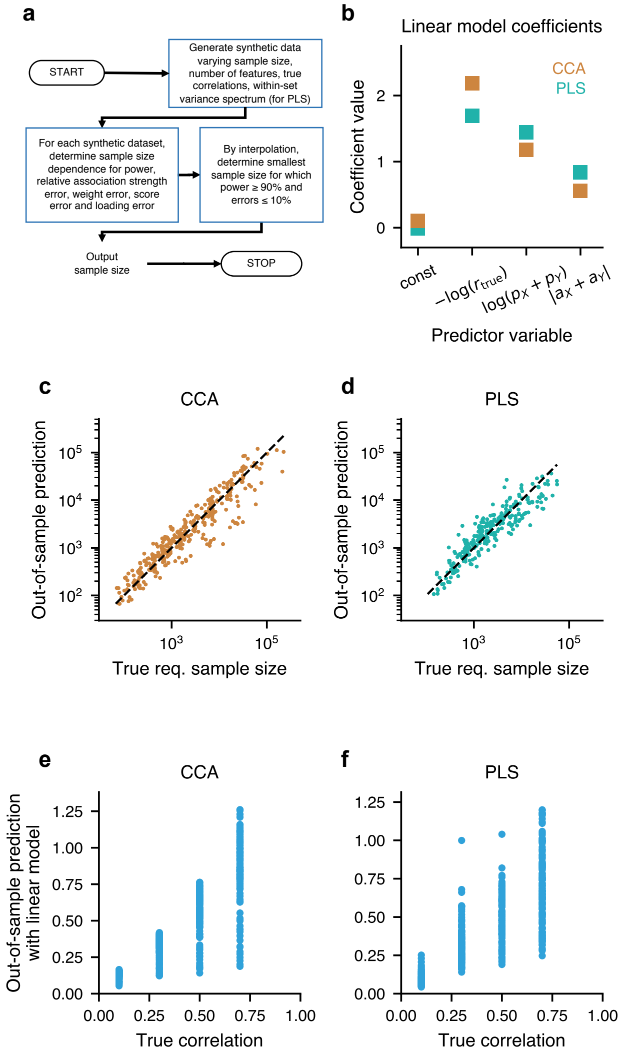

Supplementary Figure 17. Sample-size calculator. **a)** Algorithm for sample-size calculation. Sample sizes can, in principle, be calculated directly with GEMMR, as shown in Fig. 7c-d. However, this is computationally expensive. To quickly obtain sample-size estimates, we developed the algorithm illustrated here. **b-g)** Especially for low assumed ground-truth between-set correlations and a high number of features it is computationally expensive to estimate the required number of observations by generating synthetic datasets and searching the sample size such that error bounds are satisfied. To abbreviate this process we pre-calculate required sample sizes using the generative model approach for certain parameter values, fit a linear model to $\log(n_{\text{required}})$ and then use it to quickly interpolate for parameter values not in the pre-calculated database. Predictors for the linear model are $-\log(r_{\text{true}})$, $\log(p_X + p_Y)$ and, for PLS only, $|a_X + a_Y|$, where r_{true} indicates the true between-set correlation, p_X and p_Y are the number of features in datasets X and Y, respectively, and a_X and a_Y are the power-law decay constants for the within-set principal component spectrum, respectively. Shown here are linear model estimates for the required sample size based on the combined criterion, i. e. the sample sizes required to obtain 90 % power and at most 10 % error for the between-set association strength, weight, score and loading error. **b)** Linear model coefficients for CCA and PLS. **c-d)** The pre-calculated database was split in half where one half corresponded to true between-set correlations of $r_{\text{true}} = 0.1$ and 0.3, the other to $r_{\text{true}} = 0.5$ and 0.7. The linear model was re-estimated separately for each half, and used to predict the other half. We obtained good predictions for CCA (**c**) and PLS (**d**). **e-f)** Solving the linear model for r_{true}, we aim to predict between-set correlations. We train the model using either simulation outcomes for $r_{\text{true}} \in \{0.1, 0.3\}\}$, or $r_{\text{true}} \in \{0.7, 0.9\}\}$ and testing the predictions on the remaining r_{true}s. **e)** Good predictions can be obtained in this way for CCA, **f)** but not for PLS.

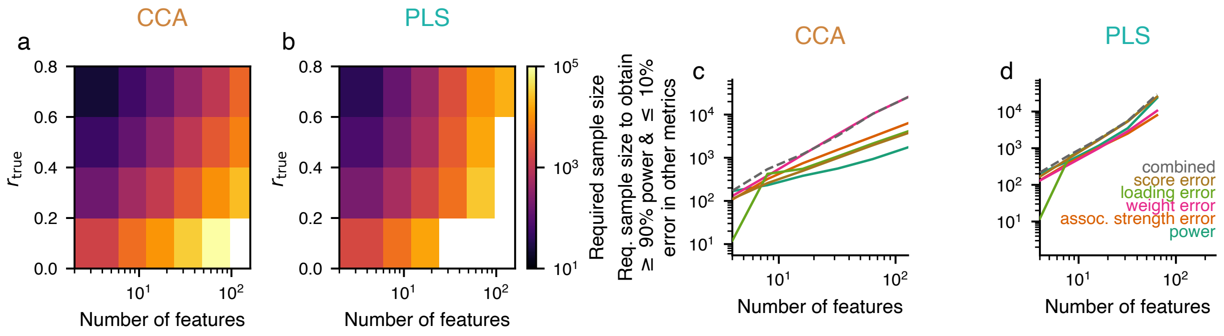

Supplementary Figure 18. Parameter dependencies of required sample sizes. a-b) Required sample sizes based on the combined criterion increase with number of features and for low true between-set correlations r_{true}. Required sample sizes were averaged across 25 generative models with different assumed weight vectors (10, due to computational expense, in the case of PLS with r_{true}=0.7 and number of features=128). Due to computational expense values for some parameter sets were not available (white). **c-d)** Scaling of sample-size dependence on number of features, shown here for $r_{\text{true}} = 0.3$, for different metrics.

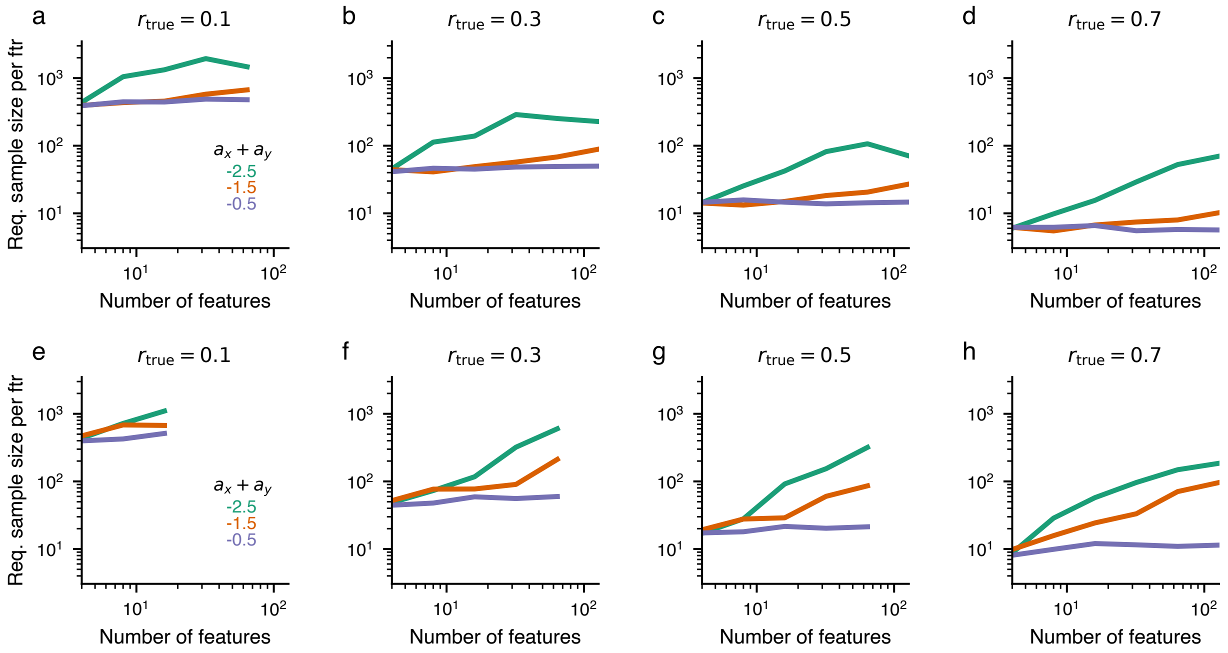

Supplementary Figure 19. Sample-size dependence on within-set variances. a-d) CCA, **e-h)** PLS. Simulated parameter sets were averaged across subsets having indicated values for the true between-set correlation r_{true} and for $a_X + a_Y$ (the sum of within-set power-law decay constants) ± 0.5. The closer $a_X + a_Y$ was to 0 (i. e. the “whiter” the data) the fewer observations were required.

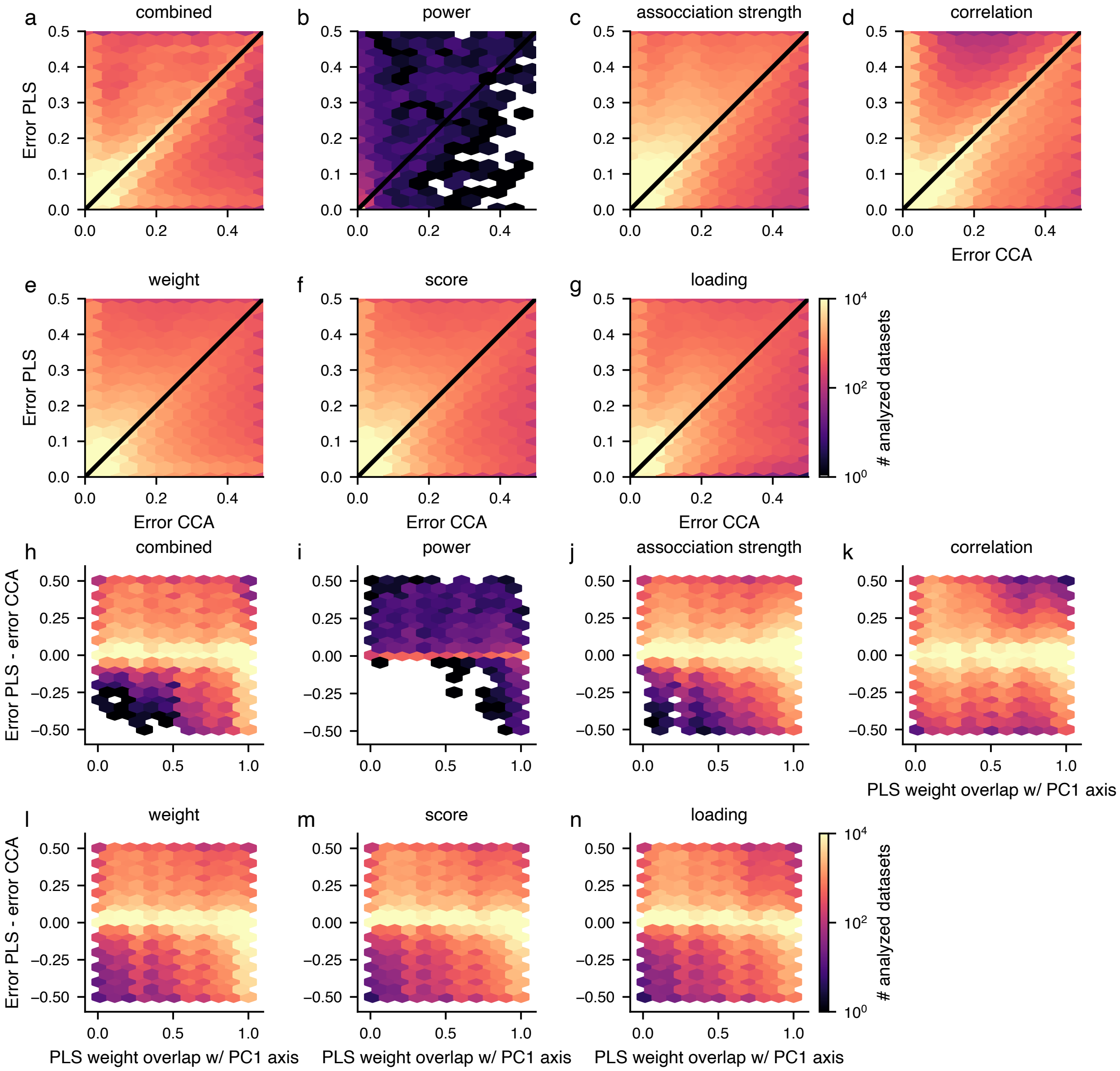

Supplementary Figure 20. Required sample size for CCA vs. PLS. We instantiated joint covariance matrices (assuming 1 between-set association mode), drew observations from the associated normal distributions, and analyzed the resulting datasets with both CCA and PLS. The CCA and PLS estimations were then compared, respectively, to the true CCA and PLS solutions, which were derived from the joint covariance matrices. Panels **a-g)** show for various error metrics how resulting deviations from the truth compare between CCA and PLS. PLS errors for a given dataset tend to be larger than CCA errors in many, but not all, datasets. **h-n)** For various error metrics, when PLS has a smaller error than CCA, this tends to happen preferentially when the true PLS weight overlaps strongly with the PC1 axis. Here, we used 25 different generative models with different assumed weight vectors (except for PLS with p_X=64 and $r = 0.7$ where we only had completed simulations for 21 completed generative models) and drew 100 collections of observations from each generative model. Datasets were included in these analyses if the CCA or PLS error were below 0.5.

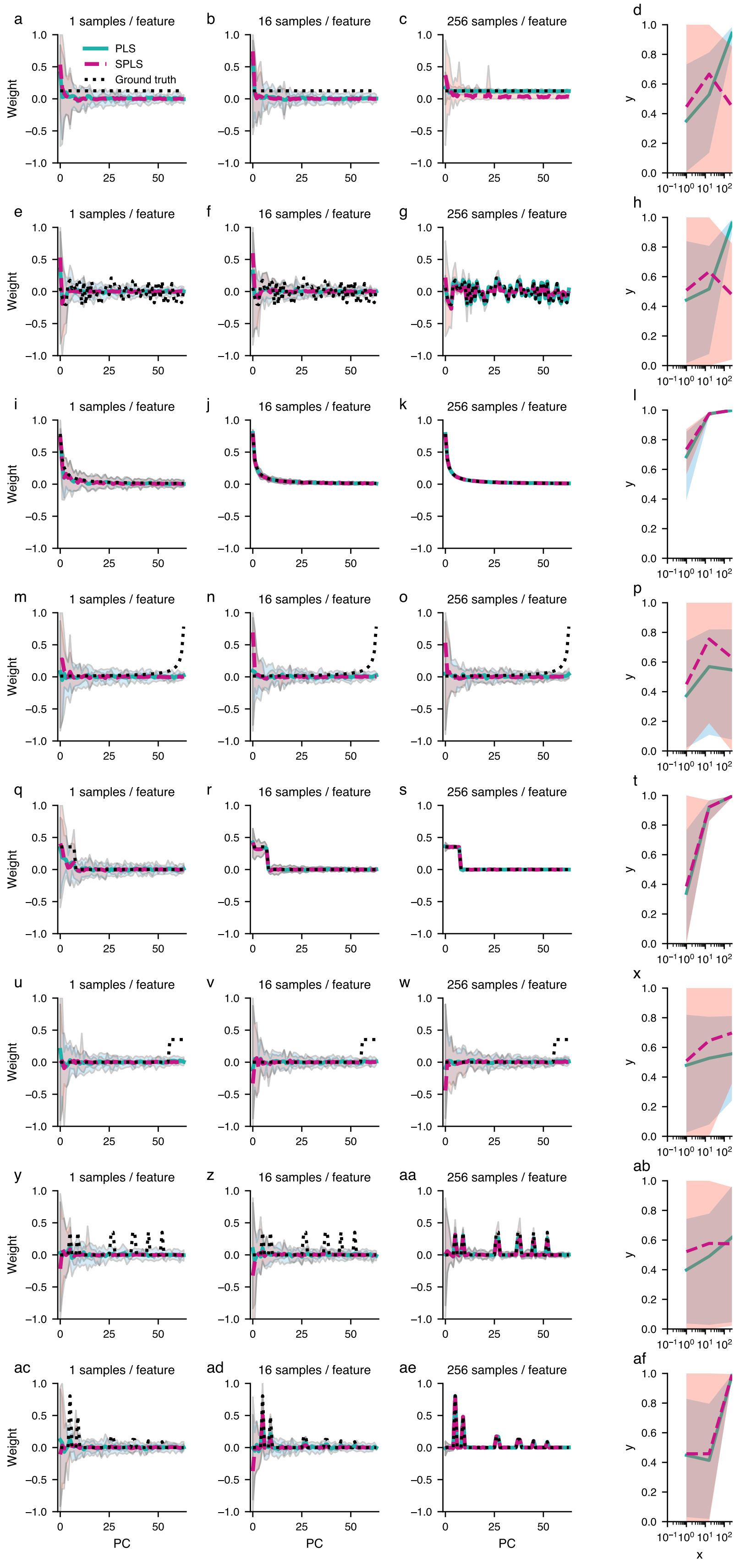

Supplementary Figure 21. Comparison of PLS and sparse PLS. (Caption follows)

Comparison of PLS and sparse CCA (PLS). We have compared the capacity of PLS and sparse PLS [Witten et al., 2009] (this method is also referred to as "sparse CCA", see Supplementary Note 3) to recover different kinds of weight profiles (cf. different rows in figure to the left). For all except the first row, PLS and sparse PLS estimated the correct weight profile provided the sample size was large enough. In the first row, where a flat weight was assumed, sparse PLS failed, although it is unclear if even larger sample sizes would allow sparse PLS to succeed. Note that, for small sample sizes, both PLS and sparse PLS put strong weight on the first principal component, irrespective of the true population weight. Note also, that weight stability (last column) was, overall, similar for PLS and sparse PLS: sparse PLS had a slight advantage for sparse population weights and small sample sizes (last 2 rows). To produce these data, we assumed the same population weight for X and Y, a population between-set correlation of 0.3 and a within-set principal component variance spectrum decaying with constant -1 (for both X and Y). We ran 10 repetitions for each parameter combination and calculated stability as the pairwise cosine similarity between all possible pairs among the 25 repetitions. Shaded areas represent 95% confidence intervals. Altogether, in the situations show-cased here, sparse PLS did not do better than PLS.

Supplementary Table 1. Considerations and recommendations for using CCA and PLS in practice.

#	Keyword	Recommendation
1.	Importance of sample size and number of features	Sample size and the number of features in the dataset are of critical importance for the stability of CCA and PLS.
2.	Significance testing	A significant non-zero association does not necessarily indicate that estimated weights are reliable.
3.	Association strength error	In-sample estimates for association strengths are too high. Out-of-sample estimates are more conservative. In-sample estimates of association strenghts should also not be taken as evidence of predictive validity of CCA/PLS model.
4.	Weights & loadings	Weights and loadings estimated with too few observations are unreliable. For PLS, estimation of cross-loadings required fewer observations than loadings.
5.	PC1 similarity	In PLS, weights can appear consistently similar to the first principal component axis.
6.	Deceptive weight stability	For PLS, weights can appear stable, scattering around the first principal component axis, and converge to their true values only for very large sample sizes.
7.	Subsampling	Subsampling can be used to check stability of estimated association strengths in empirical data: similar results for varying subsample sizes indicate stability.
8.	Reporting	Number of observations, number of features (after dimensionality reduction) and obtained association strength (in-sample and cross-validated) should be reported. The within-set variance spectrum is useful as well.
9.	Required sample size	Generally, we recommend at least 50 samples per feature for CCA, more for PLS (depending on the variance spectrum). The accompanying Python package (*GEMMR*) can be used to calculate recommended sample sizes for given dataset characteristics.

Supplementary Note 1 Canonical Correlation Analysis (CCA) and Partial Least Squares (PLS)

We assume that we have two datasets in the form of data matrices X and Y, both of which have n rows representing samples, and, respectively, p_X and p_Y columns representing measured features (or variables). Throughout we also assume that all columns of X and Y have mean 0. If both datasets consisted of only a single variable, we could measure their association by calculating their covariance or correlation. On the other hand, if one or both consist of more than one variable, pairwise between-set associations can be obtained but the possibly huge number of pairs results in a loss of statistical sensitivity and a difficulty to concisely interpret a potentially large number of significant associations [1]. To circumvent these problems, canonical correlation analysis (CCA) and partial least squares (PLS) estimate associations between weighted composites of the original data variables and find those weights that maximize the association strength.

Terminology

Given a data matrix, e. g. X, composite variables or *scores* $\vec{t}_X$ (a vector of the same size as the number of samples, n) are formed by projection of X onto a *weight* vector $\vec{w}_X$ (of same size as the number of variables in X, p_X), see Fig. 1A:

$$\vec{t}_X = X\vec{w}_X. \tag{1}$$

Loadings $\vec{\ell}_{XX}$ (of same size as the number of variables in X, p_X) characterize these composite variables by measuring their similarities with each of the original data variables in X (Supplementary Fig. 6A,B)

$$(\ell_{XX})_j = \underset{i}{\operatorname{corr}}(X_{ij}, t_{X,i}) = (\vec{x}_j)_z \cdot \left(\vec{t}_X\right)_z \tag{2}$$

where corr means Pearson correlation, $\vec{x}_j$ is the j-th column of X, and the subscript z represents z-scoring across samples (i. e. subtraction of the mean and subsequent division by the standard deviation across samples). The complete loading vector is then

$$\begin{aligned}\vec{\ell}_{XX} &= X_z^\mathsf{T} \left(\vec{t}_X\right)_z / (n-1) \\ &= \operatorname{diag}(S_{XX})^{-1/2} S_{XX}\vec{w}_X / \sqrt{\vec{w}_X^\mathsf{T} S_{XX}\vec{w}_X}\end{aligned} \tag{3}$$

where S_{XX} is the sample covariance matrix for X. Similarly, cross-loadings can be defined as

$$\begin{aligned}\vec{\ell}_{XY} &= X_z^{\mathsf{T}} \left(\vec{t}_Y\right)_z /(n-1) \\ &= \mathrm{diag}(S_{XX})^{-1/2} S_{XY} \vec{w}_Y / \sqrt{\vec{w}_Y^{\mathsf{T}} S_{YY} \vec{w}_Y}\end{aligned} \tag{4}$$

where S_{YY} and S_{XY} are, respectively, the sample covariance matrix for Y and the sample cross-covariance matrix between X and Y.

We note that alternative terminologies exist. CCA/PLS "scores" (as described above) could also be called "variates", "weights" (as described above) could also be called "vectors", and "loadings" (as described above) could also be called "parameters". For CCA, the correlation between the score vectors, i. e. the "between-set correlations" or "inter-set correlations" are also called "canonical correlations".

Partial Least Squares

Partial Least Squares (PLS) finds the maximal covariance achievable between weighted linear combinations of features from two data matrices X and Y [2]:

$$w_X, w_Y = \underset{\|\tilde{w}_X\|=1, \|\tilde{w}_Y\|=1}{\arg\max} \mathrm{cov}\left(X\tilde{w}_X, Y\tilde{w}_Y\right) \tag{5}$$

The solution is based on the between-set covariance matrix Σ_{XY} which can be estimated from data via its sampled version $S_{XY} = \frac{1}{n-1} X^{\mathsf{T}} Y$. Performing a singular value decomposition yields

$$\Sigma_{XY} = U \mathrm{diag}(\vec{\sigma}_{XY}) V^{\mathsf{T}} \tag{6}$$

such that the optimal weights are given by the first columns of U and V, and the maximal covariance

$$\max_{\|\tilde{w}_X\|=1, \|\tilde{w}_Y\|=1} \mathrm{cov}\left(X\tilde{w}_X, Y\tilde{w}_Y\right) \tag{7}$$

by the first singular value $\sigma_{XY,1}$ [3, 4].

Multiple modes of association can be estimated in this way: beyond only the first column, every pair of corresponding columns in U and V provides another mode such that $\mathrm{cov}(X\vec{u}_i, Y\vec{v}_i)$ (for $1 \leq i \leq \min(p_X, p_Y)$) is maximal given that the covariance of lower modes (those with indices $< i$) has already been accounted for. There are a number of different algorithms for PLS that differ conceptually in how these higher modes are

estimated [2, 3]. The one presented above (sometimes called “partial least squares correlation” or PLS-SVD) was chosen for its similarity to canonical correlation analysis (see below). Another notable PLS algorithm is “PLS regression” which, in contrast to the above flavor, is asymmetrical in its handling of X and Y in that it estimates weighted composites (scores) for X and re-uses these as predictors for Y [2].

Canonical Correlation Analysis

Canonical Correlation Analysis (CCA) [5], as a multivariate extension of Pearson's correlation, finds maximal correlations between weighted linear combinations of variables from X and Y:

$$\vec{w}_X, \vec{w}_Y = \underset{\tilde{\vec{w}}_X, \tilde{\vec{w}}_Y}{\arg\max} \operatorname{corr}\left(X\tilde{\vec{w}}_X, Y\tilde{\vec{w}}_Y\right) \tag{8}$$

Note that $\operatorname{corr}\left(X\tilde{\vec{w}}_X, Y\tilde{\vec{w}}_Y\right)$ is independent of the scaling of $\tilde{\vec{w}}_X$ and $\tilde{\vec{w}}_Y$. I. e. if $\vec{w}_X$ and $\vec{w}_Y$ are solutions of (8), then $c_X\vec{w}_X$ and $c_Y\vec{w}_Y$, where $c_X \in \mathbb{R}$ and $c_Y \in \mathbb{R}$, are also solutions.

Also note that, as for PLS, several modes of association can be obtained with this framework by successively discounting the variance that has been explained by lower-order modes.

The maximal correlation in (8) is often called “canonical”.

The further analysis is then based on the “whitened” between-set covariance matrix

$$\Sigma_{XY}^{(\mathrm{CCA})} = \Sigma_{XX}^{-1/2}\Sigma_{XY}\Sigma_{YY}^{-1/2} \tag{9}$$

[6, 7]. A singular value decomposition of $\Sigma_{XY}^{(\mathrm{CCA})}$ is performed, yielding

$$\Sigma_{XY}^{(\mathrm{CCA})} = U \operatorname{diag}(\vec{\sigma}_{XY}) V^{\mathsf{T}} \tag{10}$$

and the singular values $\vec{\sigma}_{XY}$ turn out to be the canonical correlations from (8), i. e. the maximal achievable correlations between a weighted linear combination of variables in X on the one hand, and a weighted linear combination of variables in Y on the other hand. The corresponding weights are given by

$$W_X = \Sigma_{XX}^{-1/2} U \tag{11}$$

$$W_Y = \Sigma_{YY}^{-1/2} V. \tag{12}$$

The use of the "whitened" between-set covariance matrix in CCA leads to an invariance property between datasets. To see this, let X_w, Y_w be whitened data matrices, i. e. $X_\mathrm{w} = X\Sigma_{XX}^{-1/2}$ and $Y_\mathrm{w} = Y\Sigma_{YY}^{-1/2}$ such that $\Sigma_{X_\mathrm{w}X_\mathrm{w}} = \mathbb{1}$, $\Sigma_{Y_\mathrm{w}Y_\mathrm{w}} = \mathbb{1}$. Then,

$$\begin{aligned}
\Sigma_{X_\mathrm{w}Y_\mathrm{w}}^{(\mathrm{CCA})} &= \Sigma_{X_\mathrm{w}X_\mathrm{w}}^{-1/2}\Sigma_{X_\mathrm{w}Y_\mathrm{w}}\Sigma_{Y_\mathrm{w}Y_\mathrm{w}}^{-1/2} &(13)\\
&= \Sigma_{X_\mathrm{w}Y_\mathrm{w}} &(14)\\
&= \Sigma_{XX}^{-1/2}\Sigma_{XY}\Sigma_{YY}^{-1/2} &(15)\\
&= \Sigma_{XY}^{(\mathrm{CCA})} &(16)
\end{aligned}$$

which is the same as for the original (non-whitened data). Consequently, canonical correlations for the original and whitened data are the same, given by the singular values of $\Sigma_{XY}^{(\mathrm{CCA})}$, canonical weights for the whitened data are directly its singular vectors and canonical weights for the original (non-whitened) data differ only by a matrix $\Sigma_{XX}^{-1/2}$ and $\Sigma_{YY}^{-1/2}$ for X and Y, respectively (see (11)-(12)).

It can be shown that the invariance property is even more general [6]. Let $O^{(X)} \in \mathbb{R}^{p_X \times p_X}$ and $O^{(Y)} \in \mathbb{R}^{p_Y \times p_Y}$ be non-singular and $\vec{d}^{(X)} \in \mathbb{R}^{p_X}$ and $\vec{d}^{(Y)} \in \mathbb{R}^{p_Y}$ be arbitrary vectors. Then $\tilde{X} = O^{(X)}X + d^{(X)}$ and $\tilde{Y} = O^{(Y)}Y + d^{(Y)}$ have the same canonical correlations as X and Y, and the canonical vectors are related by

$$\tilde{\vec{w}}_X = \left(O^{(X)}\right)^{-1}\vec{w}_X \qquad (17)$$

$$\tilde{\vec{w}}_Y = \left(O^{(Y)}\right)^{-1}\vec{w}_Y \qquad (18)$$

Thus, in particular, z-scored data $X_z = \mathrm{diag}(S_{XX})^{-1/2}X$ and $Y_z = \mathrm{diag}(S_{YY})^{-1/2}Y$ as well as whitened data X_w and Y_w have the same canonical correlations as the original data X and Y.

In CCA, X- and Y-weights are related by [8]

$$w_X = \Sigma_{XX}^{-1}\Sigma_{XY}\vec{w}_Y/\sigma_{XY} \qquad (19)$$

$$w_Y = \Sigma_{YY}^{-1}\Sigma_{YX}\vec{w}_X/\sigma_{XY} \qquad (20)$$

Replacing sample with population covariance matrices in (3) and (4), we thus also see that loadings and

cross-loadings are collinear

$$\begin{aligned}\vec{\ell}_{XY} &= \text{diag}(\Sigma_{XX})^{-1/2}\Sigma_{XY}\vec{w}_Y/\sqrt{\vec{w}_Y^\intercal\Sigma_{YY}\vec{w}_Y} \\ &= \text{diag}(\Sigma_{XX})^{-1/2}\Sigma_{XX}\Sigma_{XX}^{-1}\Sigma_{XY}\vec{w}_Y/\sqrt{\vec{w}_Y^\intercal\Sigma_{YY}\vec{w}_Y} \\ &\propto \text{diag}(\Sigma_{XX})^{-1/2}\Sigma_{XX}\vec{w}_X \\ &\propto \vec{\ell}_{XX} \end{aligned} \tag{21}$$

Overestimation of association strength

Let Σ_{XY} be a population cross-covariance matrix with singular value decomposition

$$\Sigma_{XY} = U\text{diag}(\vec{\sigma}_{XY})V^\intercal \tag{22}$$

and let $\vec{u}_1$, $\vec{v}_1$ and σ_1 be, respectively, the first columns of U, V and the first entry in $\vec{\sigma}_{XY}$. In PLS, $\vec{u}_1$, and $\vec{v}_1$ are the weight vectors of the first mode and σ_1 is the corresponding association strength.

The sample covariance matrix $S_{XY} = \frac{1}{n-1}X^\intercal Y$ is an unbiased estimator for Σ_{XY}, i. e. $\text{E}[S_{XY}] = \Sigma_{XY}$. Therefore,

$$\text{E}[\vec{u}_1^\intercal S_{XY}\vec{v}_1] = \vec{u}_1^\intercal\,\text{E}[S_{XY}]\vec{v}_1 = \vec{u}_1^\intercal\Sigma_{XY}\vec{v}_1 = \sigma_1 \tag{23}$$

i. e. if the true (but unknown) weights were applied to a given dataset (between-set covariance matrix) the association strength of the resulting scores would, on average, match the true association strength. However, by definition, PLS selects those weight vectors that maximize the association strength between resulting scores. If $\hat{\vec{u}}_1$ and $\hat{\vec{v}}_1$ are those optimal weights for a given dataset, then

$$\hat{\vec{u}}_1^\intercal S_{XY}\hat{\vec{v}}_1 \geq \vec{u}_1^\intercal S_{XY}\vec{v}_1 \tag{24}$$

and consequently also

$$\text{E}[\hat{\vec{u}}_1^\intercal S_{XY}\hat{\vec{v}}_1] \geq \text{E}[\vec{u}_1^\intercal S_{XY}\vec{v}_1] = \sigma_1 \tag{25}$$

i. e. the association strength is overestimated.

PC-1 similarity

We show here, for the special case that the between-set covariance matrix S_{XY} has rank 1, that PLS weights are more similar to the first princpial component than CCA weights.

To describe the data we choose, without loss of information, a convenient coordinate system. Specifically, we assume that both X and Y data are expressed in their respective principal component coordinate system. Now, as said, we consider here the special case that S_{XY} has rank 1, such that its singular value decomposition is

$$S_{XY} = \vec{u} s_1 \vec{v}^{\mathsf{T}}. \tag{26}$$

In this equation, $\vec{u}_{\text{PLS}} \equiv \vec{u}$ and $\vec{v}_{\text{PLS}} \equiv \vec{v}$ are, respectively, the X and Y PLS weight vectors.

To obtain the CCA solution, we consider $K = S_{XX}^{-1/2} S_{XY} S_{YY}^{-1/2}$. Then, we note that

$$K = S_{XX}^{-1/2} \vec{u} s_1 \vec{v}^{\mathsf{T}} S_{YY}^{-1/2} \tag{27}$$

$$= \frac{S_{XX}^{-1/2}\vec{u}}{\|S_{XX}^{-1/2}\vec{u}\|} \|S_{XX}^{-1/2}\vec{u}\| s_1 \|S_{YY}^{-1/2}\vec{v}\| \left(\frac{S_{YY}^{-1/2}\vec{v}}{\|S_{YY}^{-1/2}\vec{v}\|} \right)^{\mathsf{T}} \tag{28}$$

As $\left\| \frac{S_{XX}^{-1/2}\vec{u}}{\|S_{XX}^{-1/2}\vec{u}\|} \right\| = \left\| \frac{S_{YY}^{-1/2}\vec{v}}{\|S_{YY}^{-1/2}\vec{v}\|} \right\| = 1$, the right-hand side constitues the singular value decomposition of K. Thus $\vec{u}_{\text{CCA}} = \frac{S_{XX}^{-1/2}\vec{u}_{\text{PLS}}}{\|S_{XX}^{-1/2}\vec{u}_{\text{PLS}}\|}$ and $\vec{v}_{\text{CCA}} = \frac{S_{YY}^{-1/2}\vec{v}_{\text{PLS}}}{\|S_{YY}^{-1/2}\vec{v}_{\text{PLS}}\|}$ are the X and Y CCA weight vectors, respectively.

As we have assumed data are expressed in the principal component coordinate system, the cosine similarity of $\vec{u}_{\text{PLS}}$ (analogously $\vec{v}_{\text{PLS}}$) with the first principal component is simply the first coordinate of $\vec{u}_{\text{PLS}}$, $(\vec{u}_{\text{PLS}})_1$, which we denote c_{PLS}. Likewise, the cosine similarity of $\vec{u}_{\text{CCA}}$ with the first principal component is given by its first coordinate, denoted c_{CCA}. To calculate that, let $\lambda_1 \geq \cdots \geq \lambda_p$ denote the principal component variances for X and note that

$$\|S_{XX}^{-1/2}\vec{u}_{\text{PLS}}\| = \sqrt{\left(\lambda_1^{-1/2} (\vec{u}_{\text{PLS}})_1\right)^2 + \left(\lambda_2^{-1/2} (\vec{u}_{\text{PLS}})_2\right)^2 + \cdots} \tag{29}$$

$$= \lambda_1^{-1/2} \sqrt{((\vec{u}_{\text{PLS}})_1)^2 + \left(\frac{\lambda_2^{-1/2}}{\lambda_1^{-1/2}} (\vec{u}_{\text{PLS}})_2\right)^2 + \cdots} \tag{30}$$

$$\geq \lambda_1^{-1/2} \sqrt{((\vec{u}_{\text{PLS}})_1)^2 + ((\vec{u}_{\text{PLS}})_2)^2 + \cdots} \tag{31}$$

$$\geq \lambda_1^{-1/2} \|\vec{u}_{\text{PLS}}\| = \lambda_1^{-1/2} \tag{32}$$

Then,

$$c_{\text{CCA}} = \frac{\lambda_1^{-1/2}\,(\vec{u}_{\text{PLS}})_1}{\|S_{XX}^{-1/2}\vec{u}_{\text{PLS}}\|} \tag{33}$$

$$\leq \frac{\lambda_1^{-1/2} c_{\text{PLS}}}{\lambda_1^{-1/2}} \tag{34}$$

$$= c_{\text{PLS}}. \tag{35}$$

Thus, at least in the case of rank 1 between-set covariance matrices, PLS weight vectors have a stronger cosine similarity with PC1 than CCA weight vectors.

Supplementary Note 2 Scaling law for between-set correlations

When the true population canonical correlations are non-zero, the density of the observed canonical correlations is extremely complicated, following a hypergeometric function of two matrix arguments [9, Section 12.4.2].

Assuming $\Sigma_{XY} = 0$, i.e. that all population canonical correlations are 0, the density for the squares of the observed canonical correlations is given by Anderson [9, Section 13.4]. While this density is still rather complicated in general, some insight can be gained in the special case where one of the feature spaces, say X, has only 1 dimension, i.e. $p_X = 1$.

Let $p = p_X + p_Y$ and let n be the number of available samples. Then, the density d for the squares of the observed canonical corrlation, ρ_s, is [9, Section 13.4]

$$d(\rho_s) = \frac{\Gamma[\frac{1}{2}(n-1)]}{\Gamma[\frac{1}{2}(n-p)]\Gamma[\frac{1}{2}(p-1)]} \rho_s^{\frac{1}{2}(p-3)} (1-\rho_s)^{\frac{1}{2}(n-p-2)} \tag{36}$$

From this, we find the expected value of ρ_s as

$$\mathrm{E}\rho_s = \int_0^1 \rho_s d(\rho_s)\,\mathrm{d}\rho_s \tag{37}$$

$$= \frac{p-1}{n-1} \tag{38}$$

Empirically, we find this scaling law for the first (highest) observed canonical correlation to hold well for "small" n, even when $p_X > 1$, and even when the true population canonical correlation is > 0 (Supplementary Fig. 22). $p_X > 1$ leads to a vertical offset in the observed relationship, while the scaling as such appears

unchanged.

The scaling law suggests to use $(n-1)/(p-1)$ rather than the quantity "samples per feature", n/p (which we have used in multiple places throughout the manuscript). However, we do not know whether the theoretical scaling with $(n-1)/(p-1)$ would generalize to other quantities like various error metrics, beyond $\mathrm{E}\, r^2$. In fact, we have tried to re-plot Fig. 3 as a function of $(n-1)/(p-1)$ instead of n/p and this did not universally make the curves co-incide more. Moreover, "samples per feature" is conceptually simpler than $(n-1)/(p-1)$. For these reasons, we have chosen to continue to use "samples per feature" throughout the manuscript instead of $(n-1)/(p-1)$.

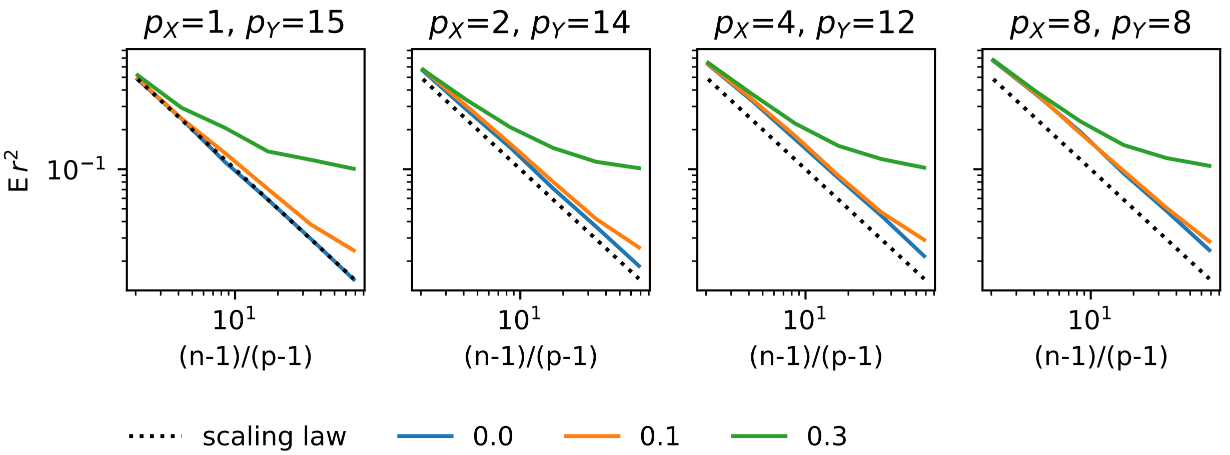

Supplementary Figure 22. Scaling of observed canonical correlation. The expected value for the square of the observed canonical correlation, $\mathrm{E}\, r^2$, largely follows the scaling law (38). Colored lines represent simulation results with different assumed true population canonical correlations. We assumed in the simulations 1 component for the between-set covariance, i.e. one true population covariance $\neq 0$. While the scaling law (black dashed curve) was derived assuming a true between-set correlation of $r_{\text{true}} = 0$ as well as assuming a dimensionality for the X feature space of $p_X = 1$, simulations show that it holds approximately for "small" n for other r_{true} (shown are $r_{\text{true}} = 0.1$ (orange) and 0.3 (green)). $p_X \neq 1$ leads to a vertical offset, while the scaling as such remains constant.

Supplementary Note 3 Sparse PLS

Multiple sparse CCA and PLS methods exist [10–13]. Here, we use *penalized matrix decomposition* (PMD) [12], which has found widespread application, see e.g. [14–19]. Briefly, the PMD algorithm repeats the following steps until convergence [12]

- $\vec{u} \leftarrow \arg\max_{\vec{u}} \vec{u}^\mathsf{T} X^\mathsf{T} Y \vec{v}$ subject to $\|\vec{u}\|_1 \leq c_1$ and $\|\vec{u}\|_2 \leq 1$

- $\vec{v} \leftarrow \arg\max_{\vec{v}} \vec{u}^\mathsf{T} X^\mathsf{T} Y \vec{v}$ subject to $\|\vec{v}\|_1 \leq c_2$ and $\|\vec{v}\|_2 \leq 1$

to maximize $\vec{u}^\mathsf{T} X^\mathsf{T} Y \vec{v}$. If $X^\mathsf{T} X \approx \mathbb{1}$ and $\|\vec{u}\|_2 = 1$, then $1 = \|\vec{u}\|_2 \approx \|X\vec{u}\|_2$ and analogously for Y. Consequently, $\vec{u}^\mathsf{T} X^\mathsf{T} Y \vec{v} \approx \vec{u}^\mathsf{T} X^\mathsf{T} Y \vec{v} / \sqrt{\|X\vec{u}\|_2 \|Y\vec{v}\|_2} = \mathrm{corr}(X\vec{u}, Y\vec{v})$. Note that the approximation $X^\mathsf{T} X \approx \mathbb{1}$ (together with $Y^\mathsf{T} Y \approx \mathbb{1}$) makes this sparse "CCA" variant identical to sparse PLS [19, 20].

Implementation and sparsity parameter selection

We implemented a Python wrapper for the R-package PMA [21] which we used with default parameters. Sparsity parameters were estimated separately for each dataset subjected to sparse CCA via 5-fold cross-validation [12, 22]: for X and Y we used 3 different candidate sparsity parameters (0.1, 0.3, and 0.5 where smaller values mean more sparsity and 1 corresponds to no sparsity), and used the same parameter for both X and Y. For each candidate parameter sparse CCA was estimated with 80 % of the data, the resulting weights applied to the remaining 20 % of the data to obtain test scores, the Pearson correlation calculated between the test scores and averaged across the 5 folds. The sparsity parameters for which the test-correlation averaged across folds was maximal, was then selected and sparse CCA re-estimated on the whole data with these parameters.

Supplementary Note 4 Reduced Rank Regression

In the following, we first derive theoretically that RRR is expected to behave similarly to CCA and PLS. We then continue to derive a generative model akin to what we have done for CCA and PLS and end with a simulation that illustrates the theoretical results.

RRR linearly predicts a p_Y-dimensional $\vec{y}$ from a p_X-dimensional $\vec{x}$, using a rank-constrained coefficient matrix C [23, Section 6.3.2]:

$$\vec{y} = C\vec{x} + Error \tag{39}$$

C is then found using a least-squares approach by minimizing [23, Section 6.3.2]:

$$W = \mathrm{E}\left[(\vec{y} - C\vec{x})^T \Gamma (\vec{y} - C\vec{x})\right] \tag{40}$$

where Γ is a positive-definite symmetric parameter matrix. Note that the choice $\Gamma = \Sigma_{YY}^{-1}$ leads to an RRR

method that is equivalent (in a specific sense) to CCA, see [23, Section 6.3.2]. The C that minimizes (40) is [23, Section 6.3.2]

$$C = \Gamma^{-\frac{1}{2}} V_q V_q^T \Gamma^{\frac{1}{2}} \Sigma_{YX} \Sigma_{XX}^{-1} \tag{41}$$

where q is the imposed rank of C, Σ_{AB} is the (cross-)covariance matrix between $\vec{a}$ and $\vec{b}$ (both assumed to be de-meaned), and the columns of $V_q = (\vec{v}_1, \ldots, \vec{v}_q)$ are the eigenvectors corresponding to the q largest eigenvalues (in descending order) of the matrix

$$R = \Gamma^{\frac{1}{2}} \Sigma_{YX} \Sigma_{XX}^{-1} \Sigma_{XY} \Gamma^{\frac{1}{2}}. \tag{42}$$

To analyze sample-size-dependence of RRR in the same way as we have done for CCC and PLS, we need a generative model that lets us simulate $\vec{x}$ and $\vec{y}$ with known RRR-solution. To that end, we note the following observations:

We first rewrite

$$C = \Gamma^{-\frac{1}{2}} \tilde{C} \Sigma_{XX}^{-\frac{1}{2}} \tag{43}$$

with

$$\tilde{C} = V_q V_q^T \Gamma^{\frac{1}{2}} \Sigma_{YX} \Sigma_{XX}^{-\frac{1}{2}} \tag{44}$$

Next, we make the following coordinate substitions:

- $\vec{x} \to \vec{x}_W = \Sigma_{XX}^{-\frac{1}{2}} \vec{x}$
- $\vec{y} \to \vec{y}_\Gamma = \Gamma^{\frac{1}{2}} \vec{y}$

Then,

$$R = \Sigma_{Y_\Gamma X_W} \Sigma_{X_W Y_\Gamma}. \tag{45}$$

Using the singular value decomposition of $\Sigma_{X_W Y_\Gamma}$,

$$\Sigma_{X_W Y_\Gamma} = U S V^T \tag{46}$$

we get

$$R = VS^2V^T. \tag{47}$$

Thus, the eigenvalues of R are the diagonal entries of S^2 and the corresponding eigenvectors are V, i. e. the right singular vectors of $\Sigma_{X_W Y_\Gamma}$. Also,

$$\tilde{C} = V_q V_q^T \Sigma_{Y_\Gamma X_W} \tag{48}$$

$$= V_q V_q^T V S U^T \tag{49}$$

$$= V_q S_q U_q^T \tag{50}$$

and

$$C = \left(\Gamma^{-\frac{1}{2}} V_q\right) S_q \left(\Sigma_{XX}^{-\frac{1}{2}} U_q\right)^T \tag{51}$$

where S_q is the top left $q \times q$ submatrix of S and U_q represents the first q columns of U.

In summary, to solve RRR, we calculate the singular value decomposition of $\Sigma_{X_W Y_\Gamma} = \Sigma_{XX}^{-\frac{1}{2}} \Sigma_{XY} \Gamma^{\frac{1}{2}} = U^{(\mathrm{RRR})} S^{(\mathrm{RRR})} \left(V^{(\mathrm{RRR})}\right)^T$, restrict $U^{(\mathrm{RRR})}$, $S^{(\mathrm{RRR})}$ and $V^{(\mathrm{RRR})}$ to the first q components, obtain the RRR "weights" $W_Y^{(\mathrm{RRR})} = \Gamma^{-\frac{1}{2}} V_q^{(\mathrm{RRR})}$ and $W_X^{(\mathrm{RRR})} = \Sigma_{XX}^{-\frac{1}{2}} U_q$, and then the RRR prediction matrix $C_q = W_Y^{(\mathrm{RRR})} S_q^{(\mathrm{RRR})} \left(W_X^{(\mathrm{RRR})}\right)^T$.

Compare this to

- PLS, where we obtained the weight vectors $W_X^{(\mathrm{PLS})}$ and $W_Y^{(\mathrm{PLS})}$ from the singular value decomposition of $I \Sigma_{XY} I = U^{(\mathrm{PLS})} S^{(\mathrm{PLS})} \left(V^{(\mathrm{PLS})}\right)^T$ as $W_X^{(\mathrm{PLS})} = I U_q^{(\mathrm{PLS})}$ and $W_Y^{(\mathrm{PLS})} = I V_q^{(\mathrm{PLS})}$
- CCA, where we obtained the weight vectors $W_X^{(\mathrm{CCA})}$ and $W_Y^{(\mathrm{CCA})}$ from the singular value decomposition of $\Sigma_{XX}^{-\frac{1}{2}} \Sigma_{XY} \Sigma_{YY}^{-\frac{1}{2}} = U^{(\mathrm{CCA})} S^{(\mathrm{CCA})} \left(V^{(\mathrm{CCA})}\right)^T$ as $W_X^{(\mathrm{CCA})} = \Sigma_{XX}^{-\frac{1}{2}} U_q^{(\mathrm{CCA})}$ and $W_Y^{(\mathrm{CCA})} = \Sigma_{YY}^{-\frac{1}{2}} V_q^{(\mathrm{CCA})}$

Thus, $\Gamma = \Sigma_{YY}$ leads to an RRR-method with the same sampling properties as CCA. $\Gamma = I$ provides a hybrid between PLS and CCA in which the X variables are internally whitened (as in CCA), but the Y variables are not (as in PLS), and the sampling properties are therefore again not different from CCA and PLS.

To illustrate this with simulations, we next derive a generative model. As we did for CCA and PLS, we will work in the coordinate system in which the within-set covariance matrices are diagonal and assume that

the diagonal entries follow a power-law. The between-set covariance matrix, Σ_{XY}, is

$$\Sigma_{XY} = \Sigma_{XX}^{\frac{1}{2}} \Sigma_{X_w Y_\Gamma} \Gamma^{-\frac{1}{2}} \tag{52}$$

$$= \Sigma_{XX}^{\frac{1}{2}} U^{(\mathrm{RRR})} S^{(\mathrm{RRR})} \left(V^{(\mathrm{RRR})}\right)^T \Gamma^{-\frac{1}{2}} \tag{53}$$

$$= \Sigma_{XX}^{\frac{1}{2}} \left(\Sigma_{XX}^{\frac{1}{2}} W_X^{(\mathrm{RRR})}\right) S^{(\mathrm{RRR})} \left(\Gamma^{\frac{1}{2}} W_Y^{(\mathrm{RRR})}\right)^T \Gamma^{-\frac{1}{2}} \tag{54}$$

As $U^{(\mathrm{RRR})}$ and $V^{(\mathrm{RRR})}$ are, respectively, left and right singular vectors, we also need to take into account the following constraints:

$$\left(U^{(\mathrm{RRR})}\right)^T U^{(\mathrm{RRR})} = I \quad \Leftrightarrow \quad \left(W_X^{(\mathrm{RRR})}\right)^T \Sigma_{XX} W_X^{(\mathrm{RRR})} = I \tag{55}$$

$$\left(V^{(\mathrm{RRR})}\right)^T V^{(\mathrm{RRR})} = I \quad \Leftrightarrow \quad \left(W_Y^{(\mathrm{RRR})}\right)^T \Gamma W_Y^{(\mathrm{RRR})} = I \tag{56}$$

We will now make the following further assumptions:

1. Σ_{XY} has rank one, such that $U^{(\mathrm{RRR})}$, $V^{(\mathrm{RRR})}$, $W_X^{(\mathrm{RRR})}$ and $W_Y^{(\mathrm{RRR})}$ all have one column
2. $\Gamma = I$

We can then choose $W_Y^{(\mathrm{RRR})}$ to be any arbitrary unit vector, set $W_X^{(\mathrm{RRR})}$ to be $\vec{w}_X / \sqrt{\vec{w}_X^T \Sigma_{XX} \vec{w}_X}$ for any arbitrary vector $\vec{w}_X$, and calculate Σ_{XY} from Eq. (54).

For CCA, the diagonal entries of $S^{(\mathrm{CCA})}$ were the between-set correlations. For PLS, the diagonal entries of $S^{(\mathrm{PLS})}$ were the between-set covariances, and, to compare PLS with CCA, we have expressed these between-set covariances as a function of the corresponding between-set correlation. This was done by multiplication with the corresponding standard deviations. To compare RRR (with $\Gamma = I$) to CCA and PLS, recall that the X and Y variables turn out to be treated similar as in CCA and PLS, respectively. Thus, by analogy, we will set the diagonal entry of $S^{(\mathrm{RRR})}$ to be the given between-set correlation multiplied by the y-standard deviations (as is done for PLS), but not by the x-standard deviations (as in CCA, the between-set correlation is not multiplied by a standard deviations, to obtain the diagonal of S).

Supplementary Fig. 23 shows results of a simulation. We have assumed that the between-set correlation is 0.3 and that the dimensionalities of the X and Y feature spaces are both 16. We have also assumed that the within-set covariances decay with a power-law with exponent -1 for both X and Y. We have then generated a random X and a random Y weight vector. For RRR we normalized these vectors according to eqs. (55) and (55), and then constructed the between-set covariance matrix according to (54). Analogously,

for PLS, we normalized both of these vectors to have length one and constructed the between-set covariance matrix according to Eq. 4 in the Methods. For CCA, on the other hand, we normalized both of these vectors according to the scheme of Eq. (55) and constructed the between-set covariance matrix according to Eq. 6 in the Methods.

We have then drawn a given number of samples (see x-axis of panels in Supplementary Fig. 23) from the associated joint normal distribution, estimated S, W_X and W_Y for PLS, CCA and RRR, and calculated a corresponding error metric. This was repeated 100 times and Supplementary Fig. 23 shows the mean of the error metrics across these 100 repetitions. Note the similarity between the convergences of PLS, CCA and RRR.

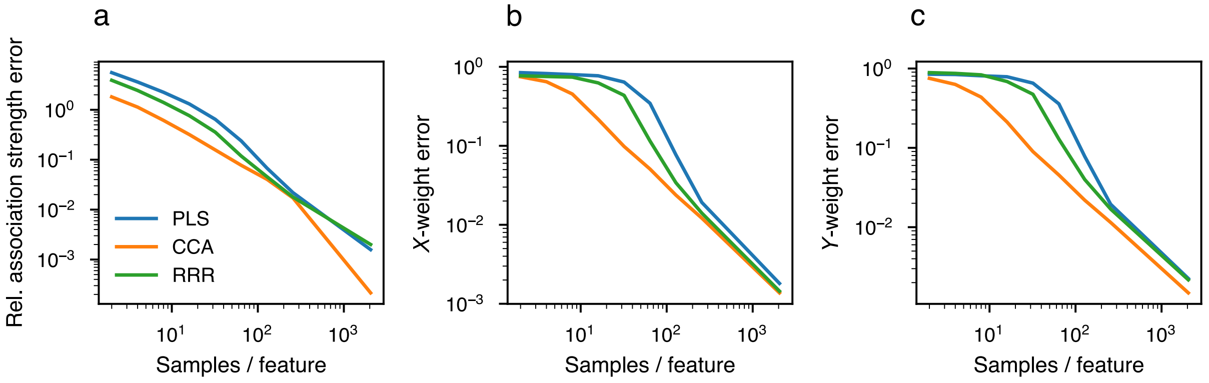

Supplementary Figure 23. Convergence of PLS, CCA and RRR. Panels show an example simulation with details described in the main text. Note the similarity between PLS, CCA and RRR. **a)** The relative association strength error is calculated as $(\hat{s} - s_{\text{true}})/s_{\text{true}}$, where $\hat{s}$ is the estimated diagonal entry of S and s_{true} is the true value that was assumed when constructing the generative model. **b, c)** The weight error is calculated as $1 - |\operatorname{cossim}(\hat{w}, w_{\text{true}})|$, where cossim denotes cosine-similarity, $\hat{w}$ is the estimated weight vector and w_{true} is the true value that was assumed when constructing the generative model.

Supplementary References

1. Smith, S. M. & Nichols, T. E. Statistical Challenges in "Big Data" Human Neuroimaging. *Neuron* **97**, 263–268 (2018).

2. Rosipal, R. & Krämer, N. Overview and Recent Advances in Partial Least Squares. In Saunders, C., Grobelnik, M., Gunn, S. & Shawe-Taylor, J. (eds.) *Subspace, Latent Structure and Feature Selection*, Lecture Notes in Computer Science, 34–51 (Springer Berlin Heidelberg, 2006).

3. Wegelin, J. A. A survey of Partial Least Squares (PLS) methods, with emphasis on the two-block case. *University of Washington, Department of Statistics, Tech. Rep* (2000).

4. Abdi, H. & Williams, L. J. Partial Least Squares Methods: Partial Least Squares Correlation and Partial Least Square Regression. In Reisfeld, B. & Mayeno, A. N. (eds.) *Computational Toxicology*, vol. 930, 549–579 (Humana Press, Totowa, NJ, 2013).

5. Hotelling, H. Relations Between Two Sets of Variates. *Biometrika* **28**, 321–377 (1936).

6. Mardia, K. V., Kent, J. T. & Bibby, J. M. *Multivariate analysis* (Academic Press, 1979), 10 edn.

7. Härdle, W. K. & Simar, L. *Applied Multivariate Statistical Analysis* (Springer International Publishing, Cham, 2019).

8. Uurtio, V. *et al.* A Tutorial on Canonical Correlation Methods. *ACM Computing Surveys (CSUR)* **50**, 95:1–95:33 (2017).

9. Anderson, T. W. *An introduction to multivariate statistical analysis*. Wiley series in probability and statistics (Wiley-Interscience, Hoboken, N.J, 2003), 3rd ed edn.

10. Lê Cao, K.-A., Rossouw, D., Robert-Granié, C. & Besse, P. A Sparse PLS for Variable Selection when Integrating Omics Data. *Statistical Applications in Genetics and Molecular Biology* **7** (2008).

11. Parkhomenko, E., Tritchler, D. & Beyene, J. Sparse Canonical Correlation Analysis with Application to Genomic Data Integration. *Statistical Applications in Genetics and Molecular Biology* **8**, 1–34 (2009).

12. Witten, D. M., Tibshirani, R. & Hastie, T. A penalized matrix decomposition, with applications to sparse principal components and canonical correlation analysis. *Biostatistics* **10**, 515–534 (2009).

13. Tenenhaus, A. *et al.* Variable selection for generalized canonical correlation analysis. *Biostatistics* **15**, 569–583 (2014).

14. Avants, B. B., Cook, P. A., Ungar, L., Gee, J. C. & Grossman, M. Dementia induces correlated reductions in white matter integrity and cortical thickness: A multivariate neuroimaging study with sparse canonical correlation analysis. *NeuroImage* **50**, 1004–1016 (2010).

15. Mizutani, S., Pauwels, E., Stoven, V., Goto, S. & Yamanishi, Y. Relating drug–protein interaction network with drug side effects. *Bioinformatics* **28**, i522–i528 (2012).

16. Yahata, N. *et al.* A small number of abnormal brain connections predicts adult autism spectrum disorder. *Nature Communications* **7**, 1–12 (2016). Number: 1 Publisher: Nature Publishing Group.

17. Xia, C. H. *et al.* Linked dimensions of psychopathology and connectivity in functional brain networks. *Nature Communications* **9**, 3003 (2018).

18. Stuart, T. *et al.* Comprehensive Integration of Single-Cell Data. *Cell* **177**, 1888–1902.e21 (2019). Publisher: Elsevier.

19. Mihalik, A. *et al.* Multiple Holdouts With Stability: Improving the Generalizability of Machine Learning Analyses of Brain–Behavior Relationships. *Biological Psychiatry* **87**, 368–376 (2020).

20. Zhuang, X., Yang, Z. & Cordes, D. A technical review of canonical correlation analysis for neuroscience applications. *Human Brain Mapping* hbm.25090 (2020).

21. Witten, D. & Tibshirani, R. PMA: Penalized Multivariate Analysis (2020). URL `https://CRAN.R-project.org/package=PMA`.

22. Le Floch, E. *et al.* Significant correlation between a set of genetic polymorphisms and a functional brain network revealed by feature selection and sparse Partial Least Squares. *NeuroImage* **63**, 11–24 (2012).

23. Izenman, A. *Modern Multivariate Statistical Techniques Regression, Classification, and Manifold Learning.* Springer Texts in Statistics (Springer, 2008).
