# Supplementary material for: On the stability of canonical correlation analysis and partial least squares with application to brain-behavior associations
Source: Commun Biol. 2024 Feb 21;7:217. doi: 10.1038/s42003-024-05869-4 (PMC11245620; doi:10.1038/s42003-024-05869-4)
Supplement: Supplementary file 3 — Reporting Summary [file 42003_2024_5869_MOESM3_ESM.pdf]

Corresponding author(s): Sotiropoulos, Murray

Last updated by author(s): Dec 4, 2023

Reporting Summary

Nature Portfolio wishes to improve the reproducibility of the work that we publish. This form provides structure for consistency and transparency in reporting. For further information on Nature Portfolio policies, see our Editorial Policies and the Editorial Policy Checklist.

Statistics

For all statistical analyses, confirm that the following items are present in the figure legend, table legend, main text, or Methods section.

n/a	Confirmed	
☐	☒	The exact sample size (*n*) for each experimental group/condition, given as a discrete number and unit of measurement
☒	☐	A statement on whether measurements were taken from distinct samples or whether the same sample was measured repeatedly
☐	☒	The statistical test(s) used AND whether they are one- or two-sided<br>*Only common tests should be described solely by name; describe more complex techniques in the Methods section.*
☒	☐	A description of all covariates tested
☒	☐	A description of any assumptions or corrections, such as tests of normality and adjustment for multiple comparisons
☒	☐	A full description of the statistical parameters including central tendency (e.g. means) or other basic estimates (e.g. regression coefficient) AND variation (e.g. standard deviation) or associated estimates of uncertainty (e.g. confidence intervals)
☒	☐	For null hypothesis testing, the test statistic (e.g. F, t, r) with confidence intervals, effect sizes, degrees of freedom and P value noted<br>*Give P values as exact values whenever suitable.*
☒	☐	For Bayesian analysis, information on the choice of priors and Markov chain Monte Carlo settings
☒	☐	For hierarchical and complex designs, identification of the appropriate level for tests and full reporting of outcomes
☐	☒	Estimates of effect sizes (e.g. Cohen's *d*, Pearson's *r*), indicating how they were calculated

Our web collection on statistics for biologists contains articles on many of the points above.

Software and code

Policy information about availability of computer code

Data collection	No data was collected specifically for this study.
Data analysis	A Python package named "GEMMR" was developed for the purpose of this study and is available from https://github.com/murraylab/gemmr.

For manuscripts utilizing custom algorithms or software that are central to the research but not yet described in published literature, software must be made available to editors and reviewers. We strongly encourage code deposition in a community repository (e.g. GitHub). See the Nature Portfolio guidelines for submitting code & software for further information.

Data

Policy information about availability of data

All manuscripts must include a data availability statement. This statement should provide the following information, where applicable:

- Accession codes, unique identifiers, or web links for publicly available datasets
- A description of any restrictions on data availability
- For clinical datasets or third party data, please ensure that the statement adheres to our policy

Human Connectome Project and UK Biobank datasets cannot be made publicly available due to data use agreements. Human Connectome Project and UK Biobank are available for researchers to apply for data access. The outcomes of synthetic datasets that were analyzed with CCA or PLS are available from https://osf.io/8expj/.

Research involving human participants, their data, or biological material

Policy information about studies with human participants or human data. See also policy information about sex, gender (identity/presentation), and sexual orientation and race, ethnicity and racism.

Reporting on sex and gender	No data was collected specifically for this study. We reused data previously collected by the Human Connectome Project (https://www.humanconnectome.org) and UK Biobank (https://www.ukbiobank.ac.uk).
Reporting on race, ethnicity, or other socially relevant groupings	No data was collected specifically for this study. We reused data previously collected by the Human Connectome Project (https://www.humanconnectome.org) and UK Biobank (https://www.ukbiobank.ac.uk).
Population characteristics	All subjects were taken from the general population.
Recruitment	No data was collected specifically for this study. We reused data previously collected by the Human Connectome Project (https://www.humanconnectome.org) and UK Biobank (https://www.ukbiobank.ac.uk).
Ethics oversight	No data was collected specifically for this study. We reused data previously collected by the Human Connectome Project (https://www.humanconnectome.org) and UK Biobank (https://www.ukbiobank.ac.uk).

Note that full information on the approval of the study protocol must also be provided in the manuscript.

Field-specific reporting

Please select the one below that is the best fit for your research. If you are not sure, read the appropriate sections before making your selection.

☒ Life sciences ☐ Behavioural & social sciences ☐ Ecological, evolutionary & environmental sciences

For a reference copy of the document with all sections, see nature.com/documents/nr-reporting-summary-flat.pdf

Life sciences study design

All studies must disclose on these points even when the disclosure is negative.

Sample size	No data was collected for this study.
Data exclusions	All subjects with sufficient scan quality were included. For the HCP fMRI vs behavior analysis, 3 subjects were not considered by PALM and dropped for this reason.
Replication	We show results for 3 different datasets.
Randomization	No data was collected for this study.
Blinding	No data was collected for this study.

Reporting for specific materials, systems and methods

We require information from authors about some types of materials, experimental systems and methods used in many studies. Here, indicate whether each material, system or method listed is relevant to your study. If you are not sure if a list item applies to your research, read the appropriate section before selecting a response.

Materials & experimental systems

n/a	Involved in the study	
☒	☐	Antibodies
☒	☐	Eukaryotic cell lines
☒	☐	Palaeontology and archaeology
☒	☐	Animals and other organisms
☒	☐	Clinical data
☒	☐	Dual use research of concern
☒	☐	Plants

Methods

n/a	Involved in the study	
☒	☐	ChIP-seq
☒	☐	Flow cytometry
☐	☒	MRI-based neuroimaging

Magnetic resonance imaging

Experimental design

Design type	Resting-state
Design specifications	HCP fMRI data comprised 2 (69 subjects), 3 (12 subjects), or 4 (870 subjects) sessions of 15 minutes each. HCP dMRI data was collected in 6 segments of roughly 9 min each. UK Biobank fMRI data comprised 1 session of 6:10 minutes.
Behavioral performance measures	No data was collected specifically for this study. We reused data previously collected by the Human Connectome Project (https://www.humanconnectome.org) and UK Biobank (https://www.ukbiobank.ac.uk).

Acquisition

Imaging type(s)	functional, structural, diffusion
Field strength	3T
Sequence & imaging parameters	No data was collected specifically for this study. We refer to descriptions of the collected data by the Human Connectome Project (van Essen et al. [2013], Neuroimage, DOI:10.1016/j.neuroimage.2013.05.041) and UK Biobank (Miller et al. [2016], Nature Neuroscience, DOI:10.1038/nn.4393)
Area of acquisition	Whole brain

Diffusion MRI ☒ Used ☐ Not used

Parameters: No data was collected specifically for this study. We refer to descriptions of the collected data by the Human Connectome Project (van Essen et al. [2013], Neuroimage, DOI:10.1016/j.neuroimage.2013.05.041)

Preprocessing

Preprocessing software	Preprocessing is described in the "Experimental Design", "Human Connectome Project (HCP) data" and "UK Biobank (UKB) data" sections of the "Methods".
Normalization	*If data were normalized/standardized, describe the approach(es): specify linear or non-linear and define image types used for transformation OR indicate that data were not normalized and explain rationale for lack of normalization.*
Normalization template	*Describe the template used for normalization/transformation, specifying subject space or group standardized space (e.g. original Talairach, MNI305, ICBM152) OR indicate that the data were not normalized.*
Noise and artifact removal	*Describe your procedure(s) for artifact and structured noise removal, specifying motion parameters, tissue signals and physiological signals (heart rate, respiration).*
Volume censoring	*Define your software and/or method and criteria for volume censoring, and state the extent of such censoring.*

Statistical modeling & inference

Model type and settings	*Specify type (mass univariate, multivariate, RSA, predictive, etc.) and describe essential details of the model at the first and second levels (e.g. fixed, random or mixed effects; drift or auto-correlation).*
Effect(s) tested	*Define precise effect in terms of the task or stimulus conditions instead of psychological concepts and indicate whether ANOVA or factorial designs were used.*

Specify type of analysis: ☐ Whole brain ☐ ROI-based ☐ Both

Statistic type for inference (See Eklund et al. 2016)	*Specify voxel-wise or cluster-wise and report all relevant parameters for cluster-wise methods.*
Correction	*Describe the type of correction and how it is obtained for multiple comparisons (e.g. FWE, FDR, permutation or Monte Carlo).*

Models & analysis

n/a	Involved in the study	
☐	☒	Functional and/or effective connectivity
☒	☐	Graph analysis
☐	☒	Multivariate modeling or predictive analysis

Functional and/or effective connectivity	Functional connectivity for the HCP - fMRI data was computed as Pearson correlations. Functional connectivity for UKB - fMRI data was computed as partial correlations.
Multivariate modeling and predictive analysis	Dimensionality reduction with principal component analysis was applied to all datasets. Data were finally analyzed with Canonical Correlation Analysis (CCA) and Partial Least Squares (PLS).
